# Evidence-Tiered Venomics of the BotG50 Toxic Fraction from *Buthus tunetanus* Reveals a Diversified Neurotoxin Repertoire

**DOI:** 10.3390/ijms27198876

**Published:** 2026-10-06

**Authors:** Rym ElFessi, Rahma Ben Abderrazek, Urvashi Khurana, Thierry Pourcher, Riadh Kharrat, Saïd Bendahhou, Riadh Marrouchi

**Affiliations:** 1 Laboratory of Venoms and Therapeutic Biomolecules, Institut Pasteur de Tunis, Université de Tunis El Manar, 13 Place Pasteur, BP74, Tunis 1002, Tunisia; riadh.kharrat@pasteur.rns.tn (R.K.); riadh.marrouchi@pasteur.tn (R.M.); 2 Laboratory of Biomolecules, Venoms and Theranostic Applications (LBVAT), Institut Pasteur de Tunis, Université de Tunis El Manar, 13 Place Pasteur, BP74, Tunis 1002, Tunisia; rahma.benabderrazek@pasteur.rns.tn; 3 Laboratoire de PhysioMédecine Moléculaire (LP2M), UMR 7370, CNRS, Université Côte d’Azur, 06107 Nice, France; ukhurana01@gmail.com; 4 Laboratory Transporter in Imaging and Radiotherapy in Oncology (TIRO), Direction de la Recherche Fondamentale (DRF), Institut des Sciences du Vivant Frédéric Joliot, Commissariat à l’Énergie Atomique et aux Énergies Alterna-tives (CEA), Université Côte d’Azur, 06107 Nice, France; thierry.pourcher@univ-cotedazur.fr

**Keywords:** scorpion venomics, nanoLC-MS/MS, Casanovo de novo sequencing, post-translational modifications, ion-channel toxins

## Abstract

Scorpion venoms represent complex pharmacological arsenals whose complete molecular inventory remains incompletely defined, particularly for North African buthid species of high medical relevance. *Buthus tunetanus* is among the most clinically significant scorpion species across the Maghreb. We analysed the toxic fraction BotG50, isolated by Sephadex G-50 gel-filtration, using nanoLC-HRMS/MS coupled with an evidence-tiered, five-stage bioinformatics workflow. The workflow combines Proteome Discoverer database searching, BLAST-guided homology expansion, Casanovo de novo sequencing with variant construction, signal-peptide and propeptide removal, and post-translational modification (PTM) annotation. The analysis revealed 64 components (FDR < 1%), comprising 38 multi-peptide-supported identifications (≥2 peptides) and 26 single-peptide hits, with a median sequence coverage of 33%. The dominant neurotoxic repertoire includes toxins targeting K^+^, Na^+^, and Cl^-^ channels, α/β toxins, insect- or mammal-specific neurotoxins, and putative depressant toxins, while a minor accessory compartment comprises hyaluronidase, phospholipase A_2_, lipolytic peptides, and hemocyanin-like proteins. BLAST analysis revealed strong sequence homologies with peptides from *Buthus israelis* (32.8%), *B. tunetanus* (15.6%), and *Mesobuthus eupeus* (10.9%). Approximately 52% of final identifications (33/64 proteins) required analytical stages beyond classical FASTA database searching. This proteomic atlas provides the most detailed molecular map of BotG50 to date, constituting a critical resource for antivenom epitope design, nanobody target selection, and bioactive peptide discovery.

## 1. Introduction

Scorpions (Arthropoda: Chelicerata) constitute an ancestral lineage whose evolutionary history dates back approximately 400 million years, making them one of the oldest known arachnid lineages to have colonised terrestrial environments [1,2,3]. Venom is their principal evolutionary innovation, serving as a biochemical weapon for capturing prey, defending against predators, and competing within their own species [1,4,5,6]. The molecular complexity of scorpion venom resides in its heterogeneous mixture of bioactive components, including inorganic substances, free amino acids, mucopolysaccharides, protease inhibitors, and enzymes such as proteases, phospholipases, and hyaluronidases [1,7,8,9]. Venoms are also rich in bioactive peptides and neurotoxins that exhibit remarkable affinity and selectivity for ion channels, particularly voltage-gated sodium (Nav), potassium (Kv), calcium (Cav), and chloride (ClC) channels [1,9,10].

From a public health perspective, scorpion envenomation constitutes a persistent global challenge, particularly across the tropical and subtropical regions of North Africa, the Middle East, Asia, and South America [10,11]. Worldwide, more than 1.2 million sting cases and approximately 3000 deaths are estimated annually [10,11]. Nearly all severe envenomations are attributed to species of the Buthidae family [10,11]. In Tunisia, the annual incidence has historically been estimated at approximately 20,000 cases [12], with *Buthus tunetanus* (Herbst, 1800) being one of the most frequently incriminated species in medical emergencies because of the high toxicity of its venom [12,13]. Facing this health burden, the molecular characterisation of venom components is essential not only to improve antivenom treatments but also to explore the pharmacological potential of these peptides in treating complex diseases, including cancer, chronic pain, autoimmune disorders, and infectious diseases [1,2,14,15,16].

Nevertheless, the full molecular complexity of scorpion venoms, including the sequence and post-translational diversity of their toxins, remains incompletely defined, particularly for the medically important but taxonomically under-represented North African *Buthus* species [9,16,17,18,19]. In this context, the BotG50 fraction of *Buthus tunetanus* occupies a singular position among the fractions generated by Sephadex G-50 gel filtration of the crude venom. Isolated as the second eluting fraction, it concentrates most of the venom material in the approximately 7 kDa range [13] and has been consistently identified in bio-guided fractionation studies as the fraction retaining the bulk of the venom’s lethal activity after intracerebroventricular injection in mice [13,20]. From a molecular standpoint, BotG50 is not a purified compound but a remarkably heterogeneous mixture: SDS-PAGE analysis reveals a dense and overlapping population of proteins and peptides spanning a broad molecular-weight range, with a protein profile that closely mirrors that of the crude venom itself [13,21]. This fraction is known to harbour ion-channel-active peptides with diverse pharmacological properties, making it the primary reservoir from which the neurotoxic arsenal of *B. tunetanus* has historically been mined [20,21,22,23,24,25,26,27,28].

The biological richness of BotG50 is well illustrated by the diversity of bioactive molecules that our laboratory and others have successively isolated from it. The potassium channel toxins Kbot21 and Kbot55, both purified from BotG50 by successive HPLC steps, represent structurally novel α-KTx subfamilies with sub-nanomolar affinity for voltage-gated Kv channels and potent lethal activity in mice [21,22]. Beyond potassium channel toxins, BotG50 has yielded insect-specific sodium channel toxins (BotIT1–BotIT6) [23,24], a chlorotoxin-like antiviral peptide (BotCl) active against Newcastle disease virus [25], an SKCa channel inhibitor [26], short anti-tumour peptides [27], and the non-toxic peptide Bot33 [28]. Despite these successive characterisations, the complete molecular catalogue of BotG50 has never been approached through a comprehensive, untargeted proteomic strategy. Each of these previous studies followed a classic bio-guided logic: one fraction, one activity, one molecule, which, by design, excludes from view the low-abundance, functionally redundant, or post-translationally modified components that collectively define the true pharmacological complexity of the fraction.

The aim of this study was therefore not to claim a definitive genome-backed inventory of *B. tunetanus* venom, but to build a transparent, evidence-tiered proteomic atlas of the BotG50 fraction. We applied an iterative, five-stage bioinformatics workflow integrating Proteome Discoverer database searching, BLAST-guided homology expansion, Casanovo deep learning-based de novo sequencing with variant construction [29], signal-peptide and propeptide removal for mature-form analysis, and systematic post-translational modification (PTM) annotation. The workflow is designed to maximise peptide identification depth in a taxonomically under-represented species for which reference sequence databases remain critically incomplete. Unlike approaches solely dependent on incomplete scorpion genomic databases, this strategy incorporates de novo sequencing, whose partly database-independent contribution extends the analysis of the full toxin arsenal and its post-translational landscape beyond the reference database alone. The resulting molecular characterisation provides the indispensable foundation for understanding the physiological efficacy of the venom in its entirety and for identifying new candidates for the development of precision-medicine therapeutics and next-generation antivenoms [13,15,30].

## 2. Results

### 2.1. Isolation of the Toxic Fraction BotG50

The crude venom of *B. tunetanus* was fractionated by Sephadex G-50 gel-filtration, generating four distinct fractions (Figure 1). Fraction II (BotG50) accounts for approximately 60% of the total venom mass and is responsible for nearly all of the venom’s lethal activity, with an intracerebroventricular LD50 of 82 ng [13]. This fraction was lyophilised and subjected to nanoLC-MS/MS analysis after tryptic digestion.

### 2.2. NanoLC-MS/MS Analysis and Global Proteomics Metrics

Chromatographic separation of the BotG50 tryptic digest resolved approximately 30 major peaks, eluting predominantly between 20 and 100 min, reflecting the high molecular complexity of the fraction (Figure 2). The five-stage bioinformatics pipeline (Figure 3) was applied to the resulting MS/MS spectra, yielding the following consolidated dataset: 64 identified venom components (38 multi-peptide-supported, ≥2 peptides; 26 tentative single-peptide), 288 distinct peptides, and 779 PSMs at a global FDR < 1%. These figures are dataset-level totals, in which each peptide sequence and each fragmentation spectrum is counted once across the entire Proteome Discoverer result; they are therefore not the arithmetic sum of the per-entry values in Supplementary Table S5. Summing the “# Unique Peptides” column of Table S5 returns a lower value (134), because that column includes only protein-specific peptides (i.e., those assigned uniquely to a single protein group) and omits sequences shared between homologous toxins, whereas summing the “# PSMs” column returns a higher value (1780), because shared (razor) spectra are attributed to every matching protein group rather than counted once. Sequence coverage ranged from 8% to 94%, with a median value of 33%. This depth of characterisation substantially exceeds prior single-stage venomics reports on North African Buthidae, which typically identify 20–40 proteins per venom fraction.

The incremental performance of each analytical stage is summarised in Table 1. The initial FASTA-only search yielded 94 proteins; the top-20 highest-coverage entries showed a mean sequence coverage of 45% (Table S2). Sequential filtering, sequence curation, and integration of complementary evidence transformed the initial set of 94 raw database hits into a final curated dataset of 64 components. Overall, 33 of the 64 final identifications (52%) required analytical stages beyond the initial FASTA database search. Stratifying the final dataset by evidence depth, 38 identifications were multi-peptide-supported, including 28 supported by ≥2 unique peptides, while the remaining 26 single-peptide identifications were retained as tentative pending future orthogonal confirmation.

### 2.3. Protein Identification by Database Searching and BLAST-Assisted Enrichment

The initial Proteome Discoverer search identified 94 proteins, predominantly neurotoxins and venom enzymes characteristic of Buthidae. Among these, the α-KTx 9.5 potassium-channel toxin (P84744) from *B. tunetanus* achieved the highest sequence coverage (93%). Other prominent identifications included the putative depressant toxin Tx131 (66%), the β-insect toxin BotIT4 (66%), the α-insect toxin Bot14 (59%), and the putative α-toxin Tx613 (52%). After incorporation of BLAST-derived homologues into the FASTA database, the β-chain lipolysis-activating peptide reached the highest PSM count among enzymes (185 PSMs), followed by hyaluronidase (51 PSMs) and the α-chain LAP (41 PSMs). The complete list of the 94 identified proteins is available in Supplementary Table S2.

BLAST-assisted enrichment substantially improved identifications for several key toxins. The Boma6c homologue (P60257) from *Buthus mardochei* Simon, 1878 (formerly *B. occitanus mardochei*), initially detected at 52% coverage as the putative α-toxin Tx613 (B8XGZ0), reached 76% coverage after database enrichment. Analysis of the results also showed that the majority of the identified proteins are neurotoxins that target potassium channels, such as α-KTx 9.5, α-KTx 8.1, α-KTx 19.1, α-KTx 9.8, α-KTx 16.6, α-KTx 27.1, Tx308, BuTXK-β, and BmTXK-β; neurotoxins that target sodium channels, such as Bot11, Bot1, Ac2, Bu1, OD1, MeuNaTxα-12, MeuNaTxβ-1, NaTx7, and AahP1005; and also insect-specific neurotoxins, such as Bot14, BotIT2, BotIT4, BotIT6, BjaIT, and LqhIT1a. The complete list of proteins identified after BLAST-guided database expansion is provided in Supplementary Table S3.

### 2.4. De Novo Sequencing, Variant Construction, and Coverage Rescue

Casanovo de novo sequencing of unassigned spectra generated 14,344 peptide predictions, exploited to construct custom protein variants and resolve sequence microheterogeneity. This approach delivered eight protein variants among the twenty highest-coverage entries. The integration of these variants into Proteome Discoverer searches enabled major coverage gains for several principal neurotoxins. For example, α-toxin Bot1 (P01488) increased from 34% to 48% via variant P01488_V (Q→K substitution at position 18), ultimately reaching 80% coverage after full PTM annotation (Discussion; Table S6); the α-toxin OD1 (P84646_V2), the α-insect toxin Bot14 (Q17254_V), and the β-toxin BmKAS (Q9UAC9_v2) reached 58%, 56%, and 41% coverage, respectively. The full list of variant-integrated identifications at Stage 3 is provided in Supplementary Table S4.

Importantly, not all proposed substitutions yielded improvements. The Boma6c toxin (P60257), which reached 76% coverage following BLAST enrichment, dropped to 67% in the single-variant form P60257_V, reflecting isoform misalignment, a useful signal that the proposed substitution does not necessarily represent the major form in the sample. Final resolution required the combined integration of multiple variant substitutions, exclusion of signal/propeptide regions, and extensive PTM annotation; the optimised B8XH01_v19 entry reached 93% sequence coverage with 286 PSMs. A second isoform variant from the same locus, B8XH01_v26, was independently derived from an alternative Casanovo-informed substitution set and yielded 188 PSMs, confirming that the LAP α-chain is represented by at least two co-occurring sequence variants in BotG50 and that the combined PSM contribution of both variants (474 PSMs) identifies this locus as the single most abundant protein component in the fraction. Likewise, α-KTx 9.8 (B8XH46) increased from 27% (canonical sequence) to 93% coverage (mature form), a >3-fold improvement attributable solely to the removal of signal/propeptide regions. A complete quantitative summary of coverage gains for all major variants relative to their wild-type counterparts is provided in Supplementary Table S6. The Casanovo de novo predictions, fragment-ion validation evidence, and Proteome Discoverer alignment screenshots supporting all principal variant assignments are provided in Supplementary Table S7.

### 2.5. Proteomic Profile Integrating Variants and Post-Translational Modifications

Coordinated integration of sequence variants and PTMs into the dynamic search space markedly improved peptide identification accuracy. The final dataset comprises 64 unique sequences. The neurotoxin-dominated subset ranged mainly from 2.9 to 11.1 kDa, in agreement with the structural characteristics of Buthidae neurotoxins, whereas accessory venom proteins extended up to 73.2 kDa. The Sequest HT search space included carbamidomethylation (+57.021 Da; C), oxidation (+15.995 Da; C, F, M, S, T, W), phosphorylation (+79.966 Da; S, T, Y), deamidation (+0.984 Da; N, Q, R), proline-to-pyrrolidinone (−30.011 Da; P), and methylation (+14.016 Da; C, D, E, H, I, K, L, N, Q, R, S, T).

With this refined search strategy, α-KTx 9.5 (P84744) retained 93% coverage (25 PSMs) throughout the workflow. Marked coverage increases were observed for several effectors: Boma6c (P60257_V + V2_F > A17) reached 94% (24 PSMs), the LAP variant B8XH01_v19 reached 93% (286 PSMs) and its co-variant B8XH01_v26 reached 86% coverage and 188 PSMs, and Bot1_V (P01488_V) reached 80% (10 PSMs) (Figure 4). PTM analysis revealed that chemical microheterogeneity is a defining feature of the BotG50 proteome: methylation was the most prevalent modification, particularly abundant on the LAP α-chain variants B8XH01_v19 and B8XH01_v26, of which B8XH01_v19 carries 21 putative methylation sites (site probability ≥ 0.75) alongside one putative tyrosine phosphorylation (Y7). All modification sites reported here are putative localisations, supported by collision-induced dissociation spectra and Proteome Discoverer site probabilities (≥0.75); unambiguous site assignment would require ETD/UVPD fragmentation, a synthetic-peptide reference, or targeted validation. The complete data are provided in Supplementary Table S5; the sequence coverage distribution across the 20 highest-coverage identifications is visualised in Figure 5. As illustrated, seven of the 20 entries achieved ≥80% sequence coverage, with values ranging from 94% (P60257_V + V2_F > A17) to 48% (A0A1L2FZD4 and ACJ23088.1). Eighteen of the 20 entries correspond to canonical scorpion neurotoxin families (consistent with the functional classification presented in Section 2.6), while the two lipolysis-activating peptide α-chain variants (B8XH01_v19 and B8XH01_v26) are classified as enzymatic/accessory venom components but achieve high coverage values owing to their exceptional abundance in the fraction; the colour gradient from dark green to yellow-green visually captures the heterogeneous but overall high sequence coverage achieved by the five-stage pipeline.

### 2.6. Functional Classification and Taxonomic Distribution

The 64 identified components were classified into six structural/functional families. These family assignments and the functional descriptors used throughout (e.g., “channel toxin”, “depressant toxin”, “insect-” or “mammal-specific”) are inferred from sequence homology and conserved cysteine-motif architecture; they denote predicted rather than experimentally demonstrated activities, since no direct electrophysiological or pharmacological assays were performed in this study. Neurotoxins constituted 86% of the BotG50 proteome (55/64), consistent with the predominantly ion-channel-targeting pharmacology of this fraction. Within neurotoxins, potassium channel toxins (KScTxs) represented 21.9% of the proteome (α-KTx 9.5, 9.8, 15.2, 19.2, 27.1, 8.8, 3.9; α-KTx 15.2, 8.8 and 3.9 are tentative single-peptide assignments; Table S5). Sodium channel toxins (NaScTxs) accounted for 6.2% of components, including the highly abundant NaTx7_v2 (Supplementary Table S9). The α/β family (15.6%; Supplementary Table S10) and insect/mammal-specific neurotoxins (21.9%; Supplementary Table S12) completed the neurotoxic arsenal. Putative toxins identified through de novo sequencing including depressant toxins and chloride channel blockers accounted for an additional 20.3% of identified sequences (Supplementary Table S11). The functional distribution of the 64 identified components is illustrated in Figure 6A.

Beyond neurotoxins, BotG50 contains an enzymatic and metabolic machinery comprising 9 distinct proteins (14.1%): lipolysis-activating peptides (8.8–11.2 kDa) and high-molecular-weight enzymes such as phospholipase A2 (25 kDa) and hyaluronidase (47.5 kDa) (Supplementary Table S13). The complete protein family data are provided in Supplementary Tables S8–S13 [31,32,33,34,35,36,37,38,39,40,41,42].

The taxonomic origin distribution of BLAST best-hit sequences is summarised in Table 2 and illustrated in Figure 6B. It reveals a phylogenetic signature anchored within the genus *Buthus*: 32.8% of identifications show highest homology to *B. israelis* toxins, followed by *B. tunetanus* itself (15.6%) and *Mesobuthus eupeus* (10.9%). Smaller proportions correspond to other Old-World buthid genera, indicating that the BotG50 neurotoxic arsenal, while highly specialised, relies fundamentally on an ancestral structural scaffold conserved throughout Buthidae evolution. A single non-scorpion cross-match (the plant knottin *Trifolium subterraneum*, A0A2Z6MCU4), retrieved through the shared inhibitor cystine-knot motif, was excluded from the final set and is documented in Supplementary Table S14.

### 2.7. Semi-Quantitative Considerations on Toxin Abundance

Although true label-free quantification (MS1 peak-area integration) was not performed in this study, PSM counts provide a widely accepted semi-quantitative proxy for relative protein abundance in shotgun proteomics [43,44]. PSM counts in the final dataset span more than two orders of magnitude, ranging from 1 to 286, revealing a strongly heterogeneous abundance distribution within BotG50. The four most spectrally abundant components: the lipolysis-activating peptide variants B8XH01_v19 (286 PSMs) and B8XH01_v26 (188 PSMs), the sodium-channel toxin NaTx7_v2 (229 PSMs), and the putative β-like toxin Tx814 (201 PSMs), each exceeded the median PSM count (~6) by approximately 40-fold. PSM-based estimates should be interpreted as relative abundance rankings rather than absolute concentrations, as they remain subject to ionisation-efficiency biases, peptide-length effects, and shared-peptide ambiguities.

## 3. Discussion

Scorpion envenomation remains a persistent public-health challenge across the arid belts of the Old and New Worlds, causing more than 1.2 million stings and approximately 3000 deaths annually, with children disproportionately affected in endemic regions [10,11,12]. Case-fatality rates in paediatric intensive care units in endemic regions may reach 1–3% despite antivenom availability [11]. Within this regional context, *Buthus tunetanus* has long been recognised as one of the most clinically significant species in Tunisia [12,13], and its BotG50 fraction was historically shown to concentrate the bulk of the venom’s lethal activity in classical bio-guided fractionation studies [13,20]. Yet despite this central pharmacological role, the complete molecular inventory of BotG50 had remained elusive, a gap reflecting both the inherent complexity of buthid venoms [1,2,9,16] and the limited representation of North African scorpion sequences in public protein repositories. The present work fills that gap, providing what is, to our knowledge, the most exhaustive proteomic atlas of BotG50 to date: 64 unique components spanning six structural and functional families, with sequence coverages reaching 94% and PSM counts spanning more than two orders of magnitude. The molecular masses of the identified neurotoxic peptides (2.9–11.1 kDa) fall within the canonical range for Buthidae neurotoxins [1,2,9], indicating that the lethal arsenal of this venom is built around a population of small, disulfide-stabilised peptides rather than larger protein effectors.

The methodological core of this study is the observation that classical, single-stage database searching systematically underestimates venom complexity in taxonomically under-represented species. This is not a new realisation in venomics. Historically, related North African buthid studies likewise relied on sequential bio-guided purification of individual toxic proteins rather than untargeted proteome-wide inventories [45,46]. Calvete and colleagues argued more than a decade ago that the field needed to move from descriptive cataloguing to integrated, multi-source venomics [47]. Our data give this principle a stark numerical expression: of the 64 components finally identified, only 31 were retrieved by the FASTA-only baseline search. The remaining 33 (52%) required sequential rounds of BLAST homology mining, transformer-based de novo sequencing with Casanovo [29], mature-form sequence processing, and post-translational modification (PTM) annotation. This finding is consistent with the limited representation of scorpion toxin sequences in reference databases [3,17,18,48] and highlights the need for computational strategies that can operate without a complete species-specific database [29]. The trajectory of a single toxin makes this principle vivid. Boma6c (P60257), a homologue from *Buthus mardochei*, emerged at only 52% coverage in the canonical search as the putative α-toxin Tx613, then climbed to 76% after BLAST-based database enrichment. A first Casanovo-informed single-variant attempt counter-intuitively dropped coverage back to 67%, a signal we interpret not as algorithmic failure but as a meaningful indication of isoform misalignment, since regression on coverage upon variant introduction implies that the proposed substitution does not match the dominant form actually present in the sample. Only the combined dual-variant P60257_V + V2_F > A17, validated by deamidation at R2 and oxidation at C12, finally delivered the highest sequence coverage of the entire dataset (94%; 24 PSMs). A nearly identical narrative applies to the lipolysis-activating peptide α-chain (B8XH01). This protein was identified at 44% in both the FASTA and BLAST stages, fell to 31% after a misguided variant attempt, and ultimately reached 93% (286 PSMs) as the B8XH01_v19 entry. The rescue required combined integration of multiple substitutions, signal/propeptide removal, and a remarkably rich PTM landscape, including one phosphorylation at Y7 and twenty-one methylation sites. A second variant, B8XH01_v26, independently derived from a distinct Casanovo substitution trajectory, contributed 188 PSMs. Together, the two LAP α-chain variants account for 474 PSMs, ranking this locus as the most abundant component of BotG50 by spectral count. This microheterogeneity at the sequence level appears biologically meaningful rather than a sequencing artefact. The Casanovo de novo read-outs, manual fragment-ion validation, and Proteome Discoverer alignment screenshots supporting these variant assignments are compiled in Supplementary Table S7. Crucially, each constructed variant was retained only after independent re-identification by Proteome Discoverer under the global 1% FDR (uniform criteria detailed in Table S7), which guards against de novo sequencing artefacts being propagated into the final dataset.

The PTM dimension deserves particular emphasis, because it is here that BotG50 begins to look less like a static catalogue of canonical sequences and more like a chemically diversified arsenal. Post-translational processing is now recognised as a fundamental layer of venom complexity, enabling a single gene product to generate multiple pharmacologically distinct isoforms and dramatically expanding the effective toxin space relative to the coding genome alone [9,16,19]. The putative PTM pattern reported here—including extensive methylation on lipolysis-activating peptides, tyrosine phosphorylation on classical α-toxins (Bot1 Y42; Bu1 Y43), serine phosphorylation of α-KTx 27.1 (S7), and oxidation events on cysteine residues outside disulfide bridges—has precedent in the work of Verano-Braga and colleagues, who demonstrated that the venom of *Tityus serrulatus* carries an unexpectedly dense layer of post-translational chemistry [19]. More recently, Zeng et al. showed that the short peptidome of *Olivierus martensii* (formerly *Mesobuthus/Buthus martensii*) is similarly punctuated by modifications that classical workflows overlook [8]. Putative phosphorylation of toxin tyrosine residues is particularly intriguing because these residues may lie within molecular surfaces involved in α-toxin recognition of voltage-gated sodium channels [49,50]. However, the functional consequences of the sites proposed here remain unknown and require targeted confirmation. In our hands, PTM-aware searching is not merely theoretical. The α-toxin Bot1_V climbs from 20% to 80% coverage when methylation, oxidation, and phosphorylation are jointly considered. Even more strikingly, the sodium-channel-targeting variant NaTx7_v2 was nearly invisible in the canonical search (7% coverage) and only reached 38% after PTM annotation, despite being one of the most abundant PSM contributors in BotG50 (229 PSMs).

Functionally, the composition we report fits the pharmacological signature long ascribed to *Buthus* venoms [2,9]. The neurotoxic core (86% of the proteome) is built around two complementary modalities: potassium channel scorpion toxins (KScTxs; 21.9%; Figure 6A) that block outward K^+^ currents and prolong membrane depolarisation, and the broader sodium-channel-targeting compartment combining canonical NaScTxs (6.2%) with the α/β toxin family (15.6%) and insect/mammal-specific neurotoxins (21.9%), all of which modify voltage-gated sodium channel (Nav) gating kinetics [2,9,49,50]. The classical site-3/site-4 model underlying the actions of long-chain scorpion α- and β-toxins is well established [30,49,50]. Long-chain α-toxins (61–76 residues; four disulfide bridges) bind to neurotoxin receptor site 3 on Nav channels and impair fast inactivation. By contrast, β-toxins bind to site 4 and shift the voltage dependence of activation, while KScTxs occlude the outer pore vestibule of Kv channels through a conserved functional dyad [2,9]. The combined KScTx/NaScTx pharmacology likely underlies the clinical picture observed in severe paediatric envenomations across the Maghreb (hypersalivation, fasciculations, autonomic storm, and cardiovascular instability [11,12]). Our data show that *B. tunetanus* deploys this arsenal in characteristically rich variety, with multiple isoforms of each family present simultaneously. Layered onto this neurotoxic core is a smaller but functionally critical enzymatic compartment (14.1%), most notably a venom hyaluronidase (A0A7T9L322; 25% coverage; 82 PSMs) acting as a classical spreading factor that degrades extracellular hyaluronic acid and accelerates the systemic distribution of small neurotoxins from the envenomation site [7]. The compartment also includes a phospholipase A_2_ (A0AA50AAI4) and the lipolysis-activating peptide complex, whose precise role in envenomation pathophysiology remains an open question for the field.

Read through a phylogenetic lens, the cross-species distribution of BLAST best-hits (Figure 6B) tells a coherent evolutionary story. Although venom was extracted exclusively from *B. tunetanus*, 32.8% of identifications display highest homology to peptides catalogued from *Buthus israelis*, 15.6% to *B. tunetanus* itself, 10.9% to *Mesobuthus eupeus*, and the remaining identifications distribute across *Olivierus*, *Androctonus*, *Leiurus*, *Odontobuthus*, and other Old-World Buthidae. We note, however, that several of these reference sequences derive from specimens catalogued before the taxonomic revision of the *Buthus occitanus* complex, within which former subspecies such as *B. occitanus mardochei* are now recognised as valid species (e.g., *Buthus mardochei*) [51]. Historical toxinological and database records may therefore include misidentified specimens, and the organismal labels attached to homology-based identifications should be regarded as provisional. This pattern is the proteomic echo of the deep ancestry of scorpion venoms and of structural scaffolds conserved across Buthidae, particularly the inhibitor cystine-knot motif of KScTxs and the long-chain α/β NaTx fold [1,2,9,16]. The preponderance of *B. israelis* hits should be read with caution: it reflects not biological proximity per se but rather the comparatively rich representation of *B. israelis* sequences in UniProtKB, a consequence of intensive transcriptomic sequencing efforts on Israeli buthids [52]. The observed sequence conservation provides a rationale for investigating broadly cross-reactive antivenom antibodies and VHH nanobody panels [13,53], an avenue in which our group has direct experimental experience [13].

Set against published proteomic surveys of related species, the depth achieved here is competitive. Verano-Braga and colleagues catalogued 147 venom proteins in *T. serrulatus* venom using a combined database-search and de novo sequencing PTM-aware approach [19]; Daoudi and colleagues reported 102 components in the Moroccan *B. occitanus* venom by combining top-down and bottom-up mass spectrometry [18]; and Zeng et al. catalogued the short-peptidome of *O. martensii* venom by LC-HRMS [8]. The landmark genome of *Olivierus martensii* [17], which uncovered 116 putative toxin genes, provides an independent genomic benchmark: the 64 components we identify in a single toxic fraction of a related species is broadly consistent with the expected toxin-gene diversity of buthid venoms when a comprehensive discovery pipeline is applied. Critically, the application of transformer-based de novo sequencing represents a methodological advance beyond earlier studies of this species. Whereas conventional database-dependent proteomics is fundamentally limited by the completeness of the reference FASTA, deep learning-based de novo approaches can propose peptide sequences independent of any database, enabling the characterisation of truly novel toxins and species-specific variants [29]. A detailed family-by-family comparison is provided in Supplementary Tables S8–S13.

The translational implications of the BotG50 atlas are direct and three-fold. With respect to antivenom development, the catalogue defines a component-resolved candidate antigenic repertoire for epitope-based immunogen design. Current Tunisian antivenom is produced by hyperimmunisation of horses with whole venom, a process that generates a broad but poorly characterised polyclonal IgG response [13]. By defining the dominant toxin isoforms and their degree of cross-species conservation, the present atlas provides a basis for a rational, component-resolved immunisation strategy targeting high-abundance candidate effectors such as NaTx7_v2, Tx814, α-KTx 9.5, and the Bot1 variants identified in the present work. With respect to nanobody development, the atlas identifies discrete, structurally defined candidate epitope targets for camelid immunisation campaigns. VHH nanobodies have emerged as a promising next-generation antivenom modality owing to their small size (~15 kDa), thermal stability, capacity to access cryptic epitopes inaccessible to conventional IgGs, and suitability for recombinant production [13,53]. Our group has previously demonstrated the neutralising efficacy of dromedary-derived nanobodies against BotI-like toxins from *B. tunetanus* [13], and the present molecular catalogue expands the target space for such campaigns. Finally, the atlas positions BotG50 within the expanding landscape of scorpion-derived therapeutics for ion channelopathies, chronic pain, and oncology, applications explored extensively in the field [1,2,14,15,16].

Several limitations define the scope of the present study. First, the proteome derives from a single pooled venom sample and one nanoLC-HRMS/MS acquisition; therefore, no statistical inference on individual, geographic, or seasonal variation in venom composition is possible [3,47]. Second, PSM counts are not a substitute for MS1-based label-free quantification (e.g., iBAQ, MaxLFQ) and should only guide candidate prioritisation [43]; semi-quantitative abundance estimates based on spectral counts are subject to the well-documented biases of peptide length, charge, and detectability [44]. Third, the absence of venom-gland transcriptomics prevents definitive confirmation of de novo-derived variants and mature precursor processing events; integration of transcriptomic and proteomic data remains the strongest approach for sequence validation [3,47]. Fourth, PTM localisation, especially for +14 Da methylation shifts and phosphorylation sites in multiply-modified peptides, remains provisional until confirmed by orthogonal MS strategies such as electron-transfer dissociation (ETD) or ultraviolet photodissociation (UVPD) fragmentation [19]. Fifth, functional annotation is based primarily on sequence homology and cysteine-rich toxin architecture; the resulting assignments should therefore be interpreted as predictions of probable toxin-family membership rather than direct demonstrations of ion-channel selectivity or pharmacological activity. To limit functional overinterpretation, each assignment is labelled as Predicted, Inferred or Validated in Supplementary Tables S8–S13 according to the supporting evidence, and direct electrophysiology, toxicity, and immunological assays are required for functional validation [21,22]. Finally, iterative database enrichment introduces a risk of circular evidence, which is why the present manuscript reports evidence tiers and bases biological conclusions primarily on the multi-peptide-supported subset and on toxin families consistent with the established pharmacology of BotG50 and previously characterised *Buthus tunetanus* toxins. In particular, the 26 single-peptide assignments should be regarded as an exploratory discovery layer rather than confirmed identifications, and each warrants targeted orthogonal validation by selected or parallel reaction monitoring (SRM/PRM), immunoaffinity enrichment, venom-gland transcriptomics, or synthetic-peptide reference before being treated as established venom components.

Taken together, the BotG50 proteome reported here is not merely a longer list of components: it is a chemically diversified pharmacological arsenal whose full recognition required moving beyond the conventional FASTA-search paradigm. The proteomic atlas provided here defines a candidate antigenic repertoire for next-generation antivenom and nanobody campaigns, consistent with recent reviews of scorpion toxins and their translational potential [2,13,15,16,53]; it identifies a discrete set of high-abundance effectors (the LAP α-chain variants, NaTx7_v2, Tx814, and α-KTx 9.5) as priority candidates for synthetic chemistry and recombinant production; and it positions BotG50 within the expanding landscape of scorpion-derived therapeutics for ion channelopathies, chronic pain, and oncology [1,2,14,15,16]. The path from this molecular catalogue to validated pharmacological agents remains substantial, requiring transcriptomic integration, MS1 quantification, PTM site validation, and functional characterisation, but the present atlas establishes the foundational coordinates for future work on the *Buthus tunetanus* venomic resource.

The taxonomic attribution of historical North African *Buthus* venom samples requires caution because the former *Buthus occitanus* complex has undergone substantial revision [54,55]. The archived pooled venom analysed here originated from the Beni Khedach population historically identified as *B. occitanus tunetanus*, currently treated as *Buthus tunetanus*. Because the exact venom-producing individuals were not retained as voucher specimens, their identity could not be retrospectively reassessed using the complete set of modern morphological or molecular criteria. Consequently, the taxonomic attribution reported here is supported by the documented geographic and institutional origin of the venom and by previously published representative material, but this limitation should be considered when comparing the present proteomic dataset with historical records.

## 4. Materials and Methods

### 4.1. Scorpion Venom

Archived pooled venom was obtained from scorpions originating from the Beni Khedach region of southern Tunisia and maintained within the antiscorpion antivenom production framework of the Institut Pasteur de Tunis. The source population was historically identified as *Buthus occitanus tunetanus* in earlier toxinological studies; following current taxonomic usage, it is referred to here as *Buthus tunetanus* (Herbst, 1800). A representative specimen from the same institutional venom programme was previously documented by ElFessi et al. [28] and is reproduced in Supplementary Figure S1. Because the present study was performed retrospectively on archived pooled venom, the exact venom-producing individuals were no longer available for renewed morphological examination or voucher deposition.

### 4.2. Gel-Filtration Chromatography on Sephadex G-50

Isolation of the toxic fraction BotG50 was performed by low-pressure size-exclusion chromatography adapting the Sephadex G-50 gel-filtration step of Miranda et al. [20]. Relative to the original conditions (two 3.2 × 100 cm columns in series, 0.10 M ammonium acetate pH ≈ 8.5, 70–85 mL/h), the modifications were the narrower column diameter (2.6 × 100 cm) and the lower flow rate (26 mL/h) used below. Lyophilised crude venom was dissolved in distilled water and loaded onto two Sephadex G-50 (Cytiva, Marlborough, MA, USA) columns (2.6 × 100 cm) mounted in series, pre-equilibrated with 0.1 M ammonium acetate buffer (pH 8.5). Elution was conducted at a constant flow rate of 26 mL/h, and 5 mL fractions were collected. Protein elution was monitored by absorbance at 280 nm (Beckman Coulter, Brea, CA, USA). The second eluting fraction (BotG50, Fraction II) was pooled, lyophilised, and resuspended in Milli-Q water (Merck Millipore, Molsheim, France) for downstream analysis; samples were stored at −20 °C until use.

Only this gel-filtration step was performed; the subsequent ion-exchange purification of individual neurotoxins in the original procedure (Amberlite CG-50, CM-Sephadex C-50 and DEAE-Sephadex A-50) was not applied, as only the toxic fraction was required [21,22].

### 4.3. In-Solution Tryptic Digestion

Purified BotG50 (100 μg) was dissolved in 100 mM NH_4_HCO_3_ (Sigma-Aldrich, Darmstadt, Germany). Dithiothreitol (DTT, Sigma-Aldrich) was added to 10 mM, and the sample was reduced at 55 °C for 60 min. Alkylation was performed with iodoacetamide (Sigma-Aldrich) (final concentration 55 mM) at 37 °C for 30 min in the dark. Proteins were precipitated with cold acetone (Sigma-Aldrich) overnight at −20 °C. Tryptic digestion was carried out with sequencing-grade trypsin (Promega, Madison, WI, USA) at an enzyme/substrate ratio of 1:50 (*w*/*w*; 2 μg trypsin per 100 μg protein) in a final volume of 102 μL at 37 °C overnight. The peptide solution was evaporated and resuspended in 50 μL of 0.1% formic acid in water.

### 4.4. NanoLC-HRMS Analysis

NanoLC-HRMS analysis was performed using an Ultimate 3000 nanoRSLC system (Thermo Fisher Scientific, Waltham, MA, USA) coupled to an Orbitrap Exploris 480 mass spectrometer (Thermo Fisher Scientific) equipped with an EasySpray source. Peptides (2 μL) were preconcentrated on an Acclaim PepMap 100 C18 μ-precolumn cartridge (300 μm i.d. × 5 mm, 5 μm, 100 Å, Thermo Fisher Scientific) at 10 μL/min in H_2_O/ACN/TFA (98%/2%/0.1%), then separated on an EasySpray PepMap RSLC C18 column (75 μm i.d. × 500 mm, 2 μm, 100 Å, Thermo Fisher Scientific) at 300 nL/min, with the column temperature set to 40 °C. Mobile phases were (A) 0.1% formic acid in water and (B) 0.1% formic acid in acetonitrile; acetonitrile, formic acid and TFA were LC-MS grade (Fisher Scientific, Illkirch, France), and ultrapure water was obtained from a Milli-Q system (Merck Millipore, Molsheim, France). The gradient was: 0–3 min, 2% B; 103 min, 20% B; 123 min, 32% B; 125–130 min, 90% B.

The mass spectrometer was operated at a capillary temperature of 280 °C and a spray voltage of 1500 V. MS1 spectra were acquired in the Orbitrap at 120,000 resolution (*m*/*z* 200) over *m*/*z* 375–1500, with an AGC target of 3 × 10^6^ and a maximum injection time of 25 ms. The 20 most intense precursor ions were isolated (2 *m*/*z* isolation window) and fragmented by HCD (NCE 30%). MS2 spectra were acquired in the Orbitrap at 15,000 resolution, with an AGC target of 5 × 10^5^ and a maximum injection time of 22 ms. Dynamic exclusion was enabled with an exclusion duration of 30 s after a single occurrence (*n* = 1), using a ±10 ppm mass tolerance window with isotope exclusion. The complete experimental workflow, from sample preparation to data analysis, is illustrated in Figure 3A.

### 4.5. Multi-Stage Bioinformatics Workflow

The five-stage bioinformatics pipeline applied to the MS/MS data is illustrated in Figure 3B.

#### 4.5.1. Stage 1: Proteome Discoverer Database Search

Raw MS files (.raw) were processed in Proteome Discoverer 3.2 (Thermo Fisher Scientific) using the Sequest HT search engine, after spectral conversion to .mgf with ProteoWizard 3.0 (MSConvert). Searches were performed against a custom UniProtKB reference FASTA (accessed 4 March 2026) compiled from organism- and protein-specific keyword queries (Supplementary Table S1). Mass tolerances: 10 ppm (precursor) and 0.05 Da (fragment). Trypsin specificity with up to three missed cleavages. Variable modifications: carbamidomethylation (+57.021 Da; C), oxidation (+15.995 Da; C, F, M, S, T, W), phosphorylation (+79.966 Da; S, T, Y), deamidation (+0.984 Da; N, Q, R), proline-to-pyrrolidinone (−30.011 Da; P), and methylation (+14.016 Da; C, D, E, H, I, K, L, N, Q, R, S, T). PSM validation used Percolator (strict target FDR 0.01; relaxed 0.05). Protein FDR was set to 1% (strict). The reference database was re-searched after each expansion step (Stages 2–3), with Percolator and the same target FDR (strict 0.01) re-applied at every round, so that the global 1% FDR was maintained throughout and the progressive database growth did not relax identification stringency. Detailed parameters appear in Supplementary Data S1.

#### 4.5.2. Stage 2: BLAST Alignment of Conflict Sequences

Partially identified sequences flagged by Proteome Discoverer were submitted to online BLASTp searches against UniProtKB using the UniProt BLAST web service (accessed 18 March 2026) using “sequences of conflict” as queries. Homologues meeting E-value < 0.001 and ≥60% identity over ≥60% query coverage were incorporated into an enriched custom FASTA database for re-searching.

#### 4.5.3. Stage 3: Casanovo De Novo Sequencing and Variant Construction

Unassigned MS/MS spectra were processed using Casanovo v5.1.2 (https://github.com/Noble-Lab/casanovo, accessed on 18 March 2026) [29] with default parameters (detailed in Supplementary Data S2), generating de novo peptide predictions. Casanovo outputs were aligned with unassigned Proteome Discoverer spectra to detect sequence polymorphisms. Protein variants (single or combined amino acid substitutions relative to UniProt canonical entries) were constructed from these de novo predictions and manually validated by inspection of fragment-ion series in high-quality MS/MS spectra (≥80% b/y-ion completeness). Variant designation follows UniProt nomenclature (e.g., P60257_V + V2_F > A17). Informative variants were appended to the enriched FASTA database and re-processed.

#### 4.5.4. Stage 4: Signal Peptide and Propeptide Removal

Signal peptides and propeptides annotated by UniProt as absent from the mature, secreted venom peptide were systematically removed from the corresponding FASTA entries prior to a further round of Proteome Discoverer searching. This mature-form processing step recovers the true sequence coverage of secreted toxins; the α-KTx 9.8 entry (B8XH46), for example, increased from 27% (canonical precursor sequence) to 93% (mature peptide) upon signal-peptide and propeptide trimming, a >3-fold gain attributable solely to this step.

#### 4.5.5. Stage 5: PTM Annotation and Confidence Tiering

Post-translational modification (PTM) site localisation was based on Sequest HT-assigned positions, further validated by manual inspection of diagnostic fragment ions. Only modification sites passing a Proteome Discoverer site-probability threshold of 0.75 were retained. Identifications were stratified into two confidence tiers: (i) a multi-peptide-supported core set comprising proteins supported by ≥2 tryptic peptides at FDR < 1% (with a high-stringency subset supported by ≥2 unique peptides), and (ii) a tentative set of single-peptide identifications meeting the global FDR threshold but requiring future orthogonal confirmation. Both tiers are reported in Supplementary Table S5 with explicit confidence-level annotation. The mass spectrometry proteomics data, custom FASTA database and Casanovo outputs have been deposited in the PRIDE repository under the dataset identifier PXD080452.

### 4.6. Functional Annotation and Data Deposition

Identified sequences were functionally annotated by cross-referencing with UniProtKB/Swiss-Prot and the ToxProt database. Components were classified into functional groups (e.g., α-KTx, β-KTx, sodium channel modulators, depressant toxins) based on sequence homology and cysteine-rich motif architecture. Functional evidence levels were assigned as follows: Predicted, when the annotation relied on sequence similarity, cysteine-framework architecture, or database classification alone; Inferred, when the assignment was additionally supported by a closely related experimentally characterised homologue and conserved determinants associated with the reported activity; and Validated, when direct functional evidence was available for the same toxin or an identical mature sequence. These levels describe confidence in functional assignment and are distinct from the proteomic identification tiers used for peptide-spectrum evidence; they were never derived from, and must not be equated with, the number of peptides, the number of PSMs, or the FDR. The MS datasets and custom FASTA reference have been deposited in the PRIDE repository (ProteomeXchange) under dataset identifier PXD080452.

## 5. Conclusions

Using an iterative five-stage proteomics workflow, we catalogued 64 venom components from the BotG50 fraction of *Buthus tunetanus*. The dataset comprises 38 multi-peptide-supported identifications (≥2 peptides), of which 28 are further supported by ≥2 unique peptides and form the most stringent core for biological interpretation, together with 26 tentative single-peptide leads. Identifications are supported by 288 distinct peptides and 779 PSMs at FDR < 1%, with sequence coverages spanning 8% to 94%. The toxin repertoire is dominated by neurotoxins (86%), notably KScTxs (21.9%), the α/β family (15.6%), and other insect/mammal-specific neurotoxins (21.9%), with enzymatic components (hyaluronidase, phospholipase A_2_, and lipolysis-activating peptides) constituting approximately 14% of the proteome. Multi-stage bioinformatics was indispensable for identifying the 52% of the final protein set that escaped the initial FASTA-only search. Extending beyond classical database searching to BLAST realignment, Casanovo de novo sequencing, mature-form sequence processing, and systematic PTM curation improved the mean sequence coverage of the top-20 highest-coverage entries from 45% (FASTA-only baseline, Table S2) to 69% (full five-stage pipeline, Table S5). The observed cross-species conservation of BotG50 toxin scaffolds with *B. israelis* and related Old-World Buthidae supports further evaluation of this atlas for the rational design of broadly cross-reactive antivenom antibodies and nanobodies. This study represents an initial proteomic atlas; biological replicates and orthogonal validation are planned in follow-up work. Future work will integrate venom-gland transcriptomics with the present proteomics dataset to derive a more complete toxin-gene catalogue for this species. A reference genome for *B. tunetanus*, currently unavailable, would further reduce the limitations associated with homology-based reference databases.

## Figures and Tables

**Figure 1:**
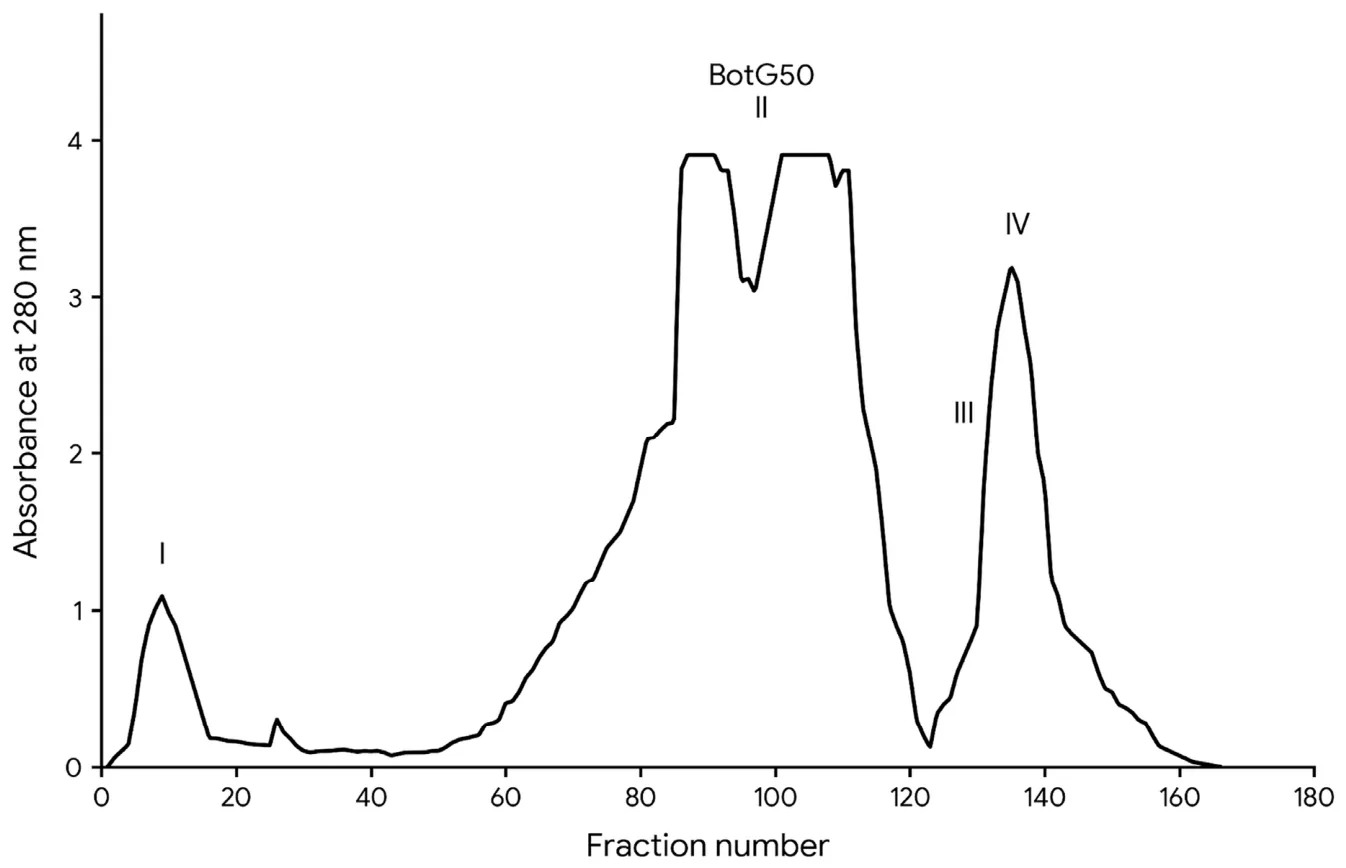
Chromatographic profile of the crude *Buthus tunetanus* venom filtration by atmospheric pressure liquid chromatography (Sephadex G-50 gel). Peak fractions are clearly resolved and labelled (I, II, III, IV) based on absorbance measurements at 280 nm.

**Figure 2:**
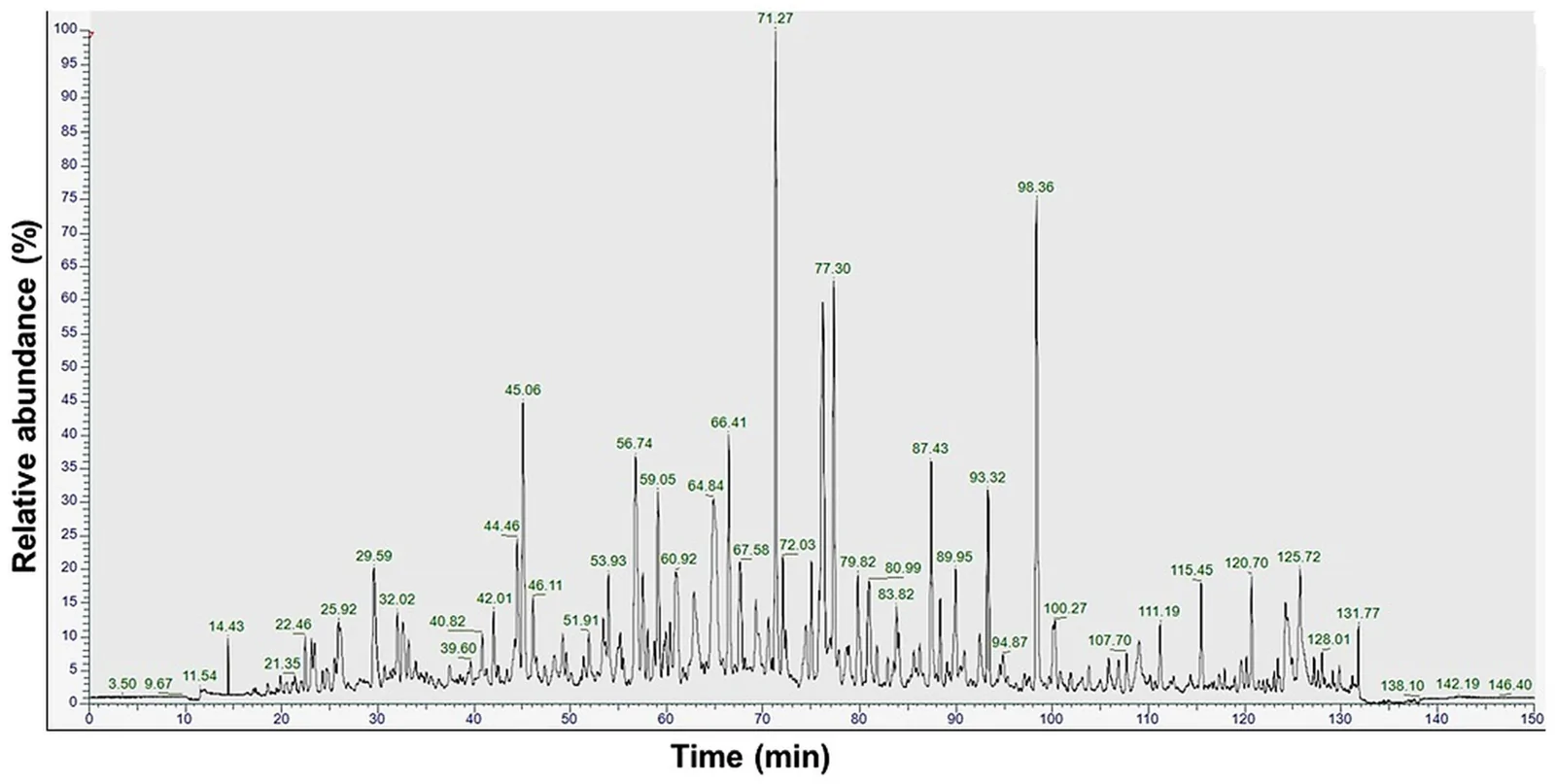
NanoLC-MS/MS total ion chromatogram (TIC) of the BotG50 fraction from *Buthus tunetanus.* Separation was achieved using a water/acetonitrile gradient containing 0.1% formic acid. Around 30 major peaks were detected, mainly eluting between 20 and 100 min, reflecting the complex composition of the fraction.

**Figure 3:**
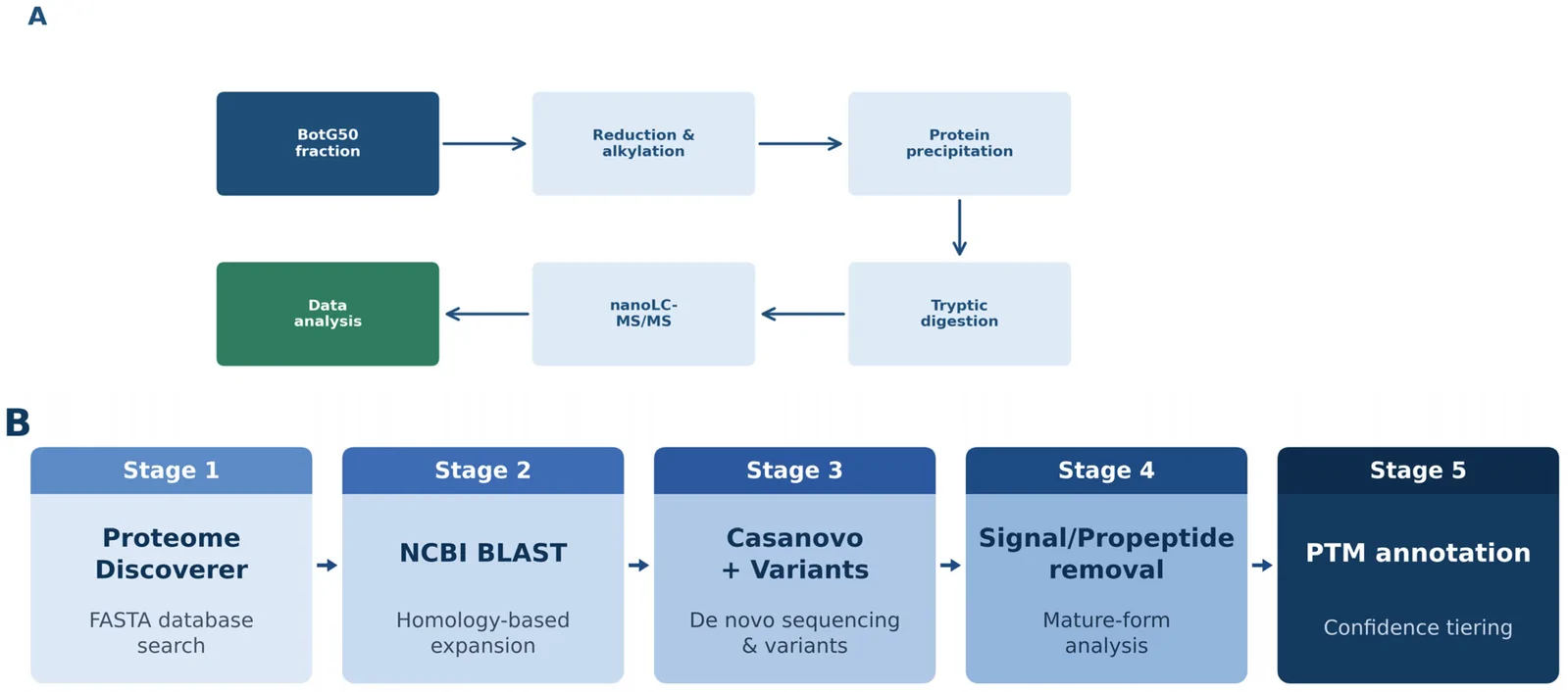
Overview of the analytical workflow applied to the BotG50 fraction from *Buthus tunetanus*. (**A**) Sample preparation pipeline: the lyophilised BotG50 fraction was resuspended, reduced and alkylated (DTT/IAA), precipitated with cold acetone, and digested overnight with sequencing-grade trypsin, prior to nanoLC-MS/MS analysis and data analysis. (**B**) Five-stage bioinformatics pipeline: Stage 1, Proteome Discoverer database search against a taxonomy-filtered custom UniProt FASTA; Stage 2, BLAST-guided homology expansion of the reference database; Stage 3, Casanovo deep-learning de novo sequencing and sequence-variant construction; Stage 4, removal of signal peptides and propeptides for mature-form analysis; Stage 5, post-translational modification (PTM) annotation and confidence tiering. The pipeline yielded a final dataset of 64 identified components, 288 distinct peptides, and 779 PSMs (FDR < 1%) with sequence coverages spanning 8–94%.

**Figure 4:**
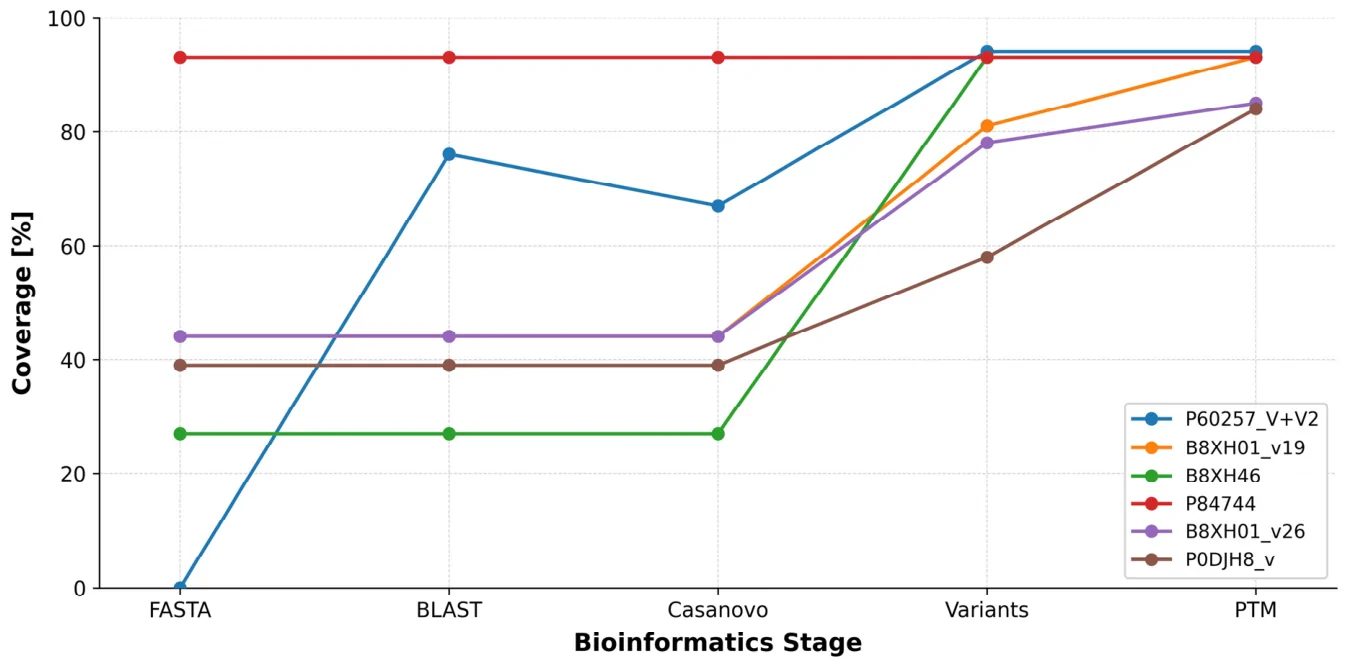
Protein sequence coverage progression through the five bioinformatics stages (Stage 1, FASTA/Proteome Discoverer → Stage 2, BLAST → Stage 3, Casanovo & Variants → Stage 4, Signal/Propeptide removal → Stage 5, PTM annotation) for six representative BotG50 toxins. P84744 (red) was fully resolved at Stage 1; P60257_V + V2 (blue) required multi-stage variant curation to reach 94% coverage; B8XH01_v19 (orange), the most abundant component in BotG50 by PSM count (286 PSMs), and its co-variant B8XH01_v26 (purple, 188 PSMs), both dropped at the single-variant step before recovering through combined substitution, propeptide removal, and PTM annotation; B8XH46 (green) and P0DJH8_v (brown) were rescued mainly at the Signal/Propeptide and PTM stages.

**Figure 5:**
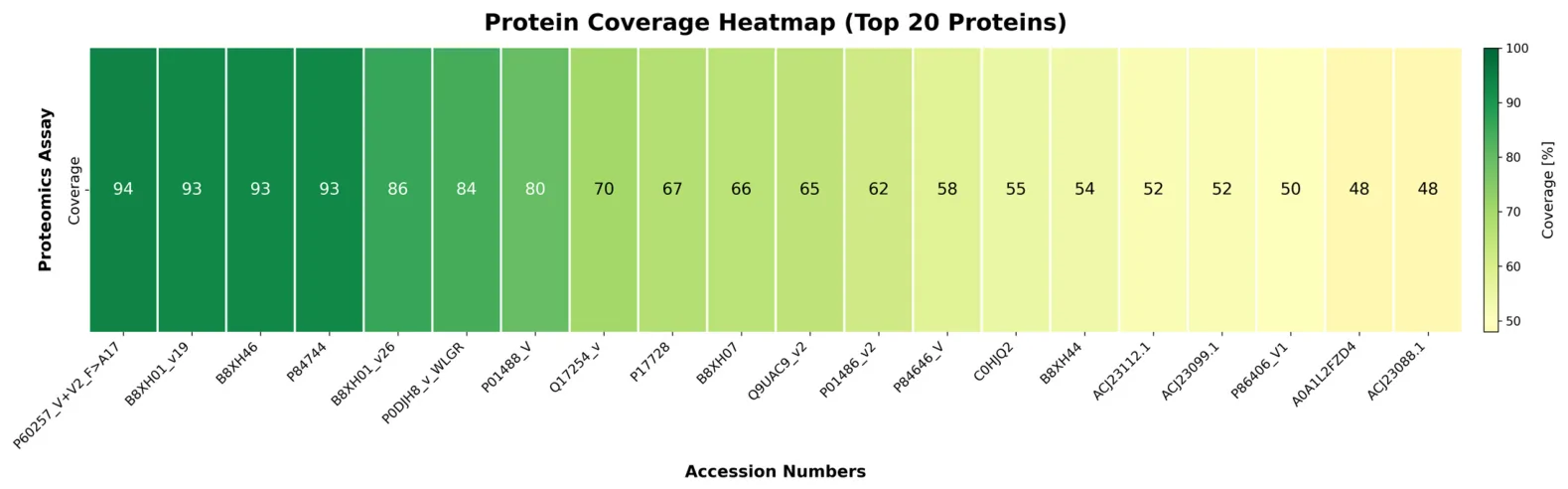
Protein sequence coverage heatmap for the top 20 identified proteins from the BotG50 fraction, ranked in decreasing order of sequence coverage. Coverage values (%) are indicated within each cell; the colour scale ranges from light yellow (low coverage) to dark green (high coverage). The four top-ranked proteins (P60257_V + V2_F > A17, B8XH01_v19, B8XH46, and P84744) achieved 94%, 93%, 93%, and 93% sequence coverage, respectively.

**Figure 6:**
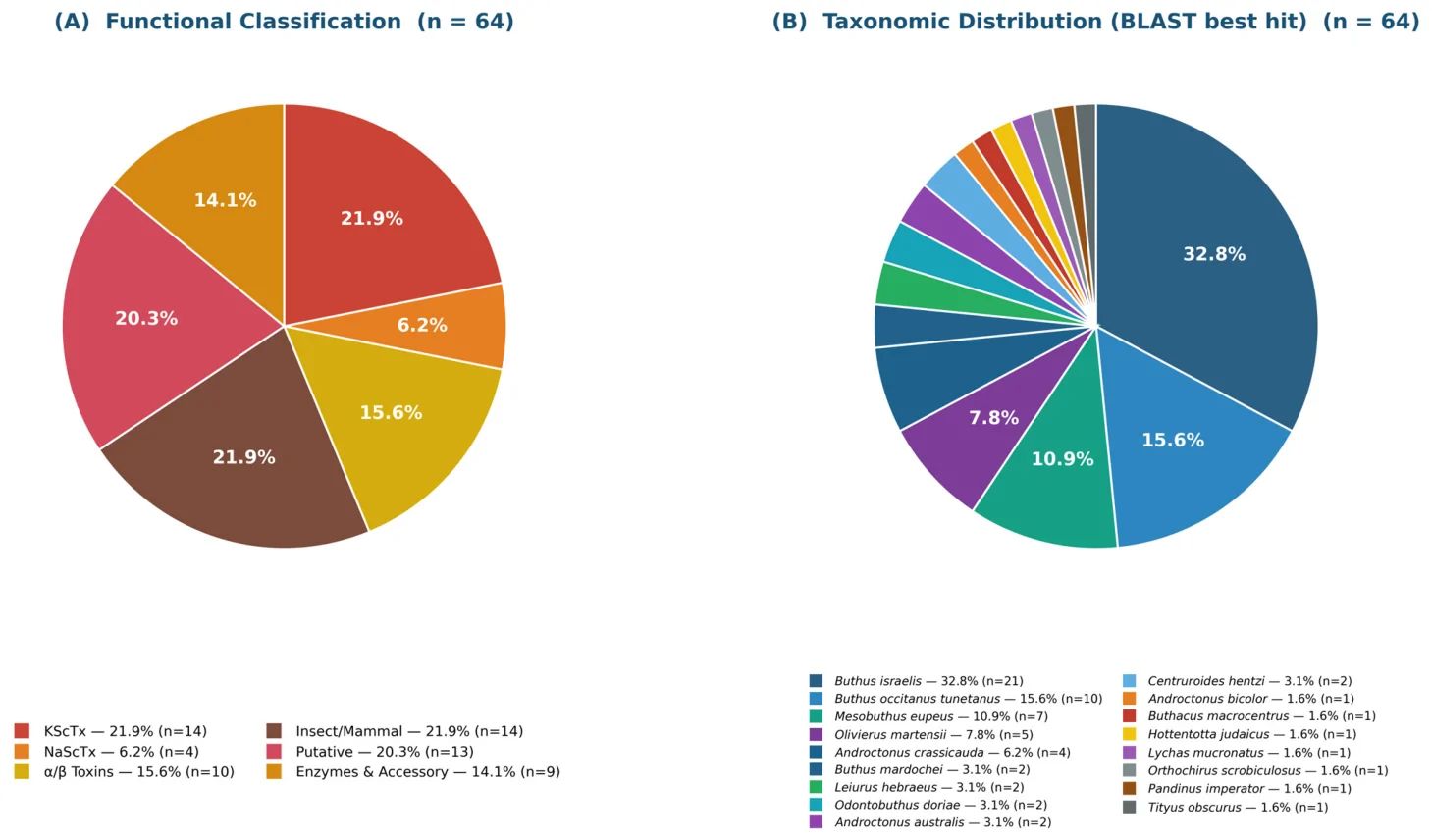
Functional and taxonomic characterisation of the BotG50 venom proteome. (**A**) Relative proportions of the six functional categories identified among the 64 BotG50 components. KScTx, potassium channel scorpion toxins; NaScTx, sodium channel scorpion toxins; α/β, alpha/beta long-chain neurotoxins; Insect/Mammal, insect- or mammal-specific excitatory and depressant neurotoxins; Putative, putative depressant and chloride channel toxins identified through de novo sequencing; Enzymes & Accessory, lipolysis-activating peptides, hyaluronidase, phospholipase A_2_ and hemocyanin-like proteins (detailed in Tables S8–S13). (**B**) Taxonomic distribution of BLAST best-hit organisms for the 64 identified proteins. The preponderance of *Buthus israelis* (32.8%) reflects the comparatively richer representation of this species in UniProtKB rather than strict phylogenetic proximity (detailed in Table 2).

**Table 1:** Incremental performance of the five-stage bioinformatics workflow applied to BotG50 proteomics data. Mean Coverage (%) is the mean sequence coverage of the top-20 highest-coverage proteins at each cumulative stage. Stages 1–5 correspond to those described in Figure 3B.

Bioinformatics Stage	Proteins (*n*)	Mean Coverage (%)	Key Addition
Stage 1: FASTA database search	94	45	FASTA database search (UniProt); 94 raw identifications (Table S2)
+Stage 2: BLAST alignment	45	42	+cross-species homologues via BLAST realignment; 45 curated proteins (Table S3)
+Stage 3: Casanovo de novo variants	39	40	+Casanovo sequence variants integrated; 39 curated proteins (Table S4)
+Stage 4: Signal/propeptide deletion	55	54	+Signal peptides and propeptides removed; 55 curated proteins with improved mature-form coverage
+ Stage 5: PTM annotation	64	69	Final curated dataset: 38 multi-peptide-supported (≥2 peptides) + 26 tentative single-peptide

Mean Coverage (%) is the mean sequence coverage of the top-20 highest-coverage proteins at each stage. Proteins (*n*) at Stage 1 reports raw FASTA-only identifications; Stages 2–4 report curated subsets after homology-based filtering (Stage 2), variant validation (Stage 3), and mature-form processing (Stage 4); Stage 5 reports the final integrated dataset combining all evidence streams (Tables S2–S5). Stage 4 mean coverage (54%) estimated from reconstructed dataset (signal/propeptide deletion applied, prior to PTM annotation). De novo sequencing at Stage 3 used Casanovo.

**Table 2:** Taxonomic distribution of BLAST best-hit organisms for the 64 venom components identified in the BotG50 fraction. *n*, number of identifications per taxon.

Organism	*n*	%
*Buthus israelis*	21	32.8
*Buthus tunetanus*	10	15.6
*Mesobuthus eupeus*	7	10.9
*Olivierus martensii*	5	7.8
*Androctonus crassicauda*	4	6.2
*Buthus mardochei*	2	3.1
*Leiurus hebraeus*	2	3.1
*Odontobuthus doriae*	2	3.1
*Androctonus australis*	2	3.1
*Centruroides hentzi*	2	3.1
*Androctonus bicolor*	1	1.6
*Buthacus macrocentrus*	1	1.6
*Hottentotta judaicus*	1	1.6
*Lychas mucronatus*	1	1.6
*Orthochirus scrobiculosus*	1	1.6
*Pandinus imperator*	1	1.6
*Tityus obscurus*	1	1.6
Total	64	100.0

## Data Availability

The mass spectrometry proteomics data, including raw files (.raw and .mgf), the custom UniProt FASTA scorpion toxin database, Proteome Discoverer search result files, and Casanovo de novo sequencing output, have been deposited to the ProteomeXchange Consortium via the PRIDE partner repository with the dataset identifier PXD080452.

## Supplementary Materials

The following supporting information can be downloaded at: https://www.mdpi.com/article/10.3390/ijms27198876/s1.
