# Supplementary material for: Evidence-Tiered Venomics of the BotG50 Toxic Fraction from Buthus tunetanus Reveals a Diversified Neurotoxin Repertoire
Source: Int J Mol Sci. 2026 Oct 6;27(19):8876. doi: 10.3390/ijms27198876 (PMC13664939; doi:10.3390/ijms27198876)
Supplement: Supplementary file 1 [file ijms-27-08876-s001.zip › ijms-4446687-supplementary.pdf]

Supplementary Materials

Evidence-Tiered Venomics of the BotG50 Toxic Fraction from Buthus tunetanus Reveals a Diversified Neurotoxin Repertoire

Rym ElFessi[1, *], Rahma Ben Abderrazek[2], Urvashi Khurana[3], Thierry Pourcher[4], Riadh Kharrat[1], Saïd Bendahhou[3, *, ‡] and Riadh Marrouchi [1, ‡]

This file contains the following tables, numbered in order of appearance and supplementary figure S1:

Table S1: Summary of UniProt database search queries used for sequence retrieval.

Table S2: Protein identifications by Proteome Discoverer 3.2 against the UniProtKB/Swiss-Prot database.

Table S3: Protein identifications after BLAST-guided alignment expansion. Bold accession numbers denote proteins newly identified at this analytical stage.

Table S4: Protein identifications including sequence variants.

Table S5: Protein identifications combining sequence variants, deleted signal-peptide/propeptide regions, BLAST-derived homologs and post-translational modifications (PTMs).

Table S6: Sequence coverage of major protein variants compared with their wild-type counterparts.

Table S7: Detailed wild-type/variant comparison, including Casanovo *de novo* sequencing results and screenshots of the regions identified by Proteome Discoverer.

Table S8: Categorization of channel toxins identified in BotG50 — Potassium channel toxins.

Table S9: Categorization of channel toxins identified in BotG50 — Sodium channel toxins.

Table S10: Categorization of channel toxins identified in BotG50 — Alpha/Beta toxins.

Table S11: Categorization of channel toxins identified in BotG50 — Putative toxins.

Table S12: Categorization of channel toxins identified in BotG50 — Insect/Mammal-specific neurotoxins.

Table S13: Categorization of channel toxins identified in BotG50 — Enzymes and accessory venom proteins.

Table S14: Excluded non-scorpion database cross-match.

Figure S1: Representative habitus of *Buthus tunetanus* from the institutional scorpion venom programme of the Institut Pasteur de Tunis. The photograph was previously published under the historical designation *Buthus occitanus tunetanus* by ElFessi et al. (2022).

Table S1. Summary of UniProt database search queries used for sequence retrieval.

S. No.	KEYWORD	Dates	UniProt result
1	Scorpionidae	4 March 2026	423
2	Scorpionidae AND toxin	4 March 2026	86
3	Scorpionidae AND venom	4 March 2026	168
4	Scorpionidae AND sting	4 March 2026	1
5	Scorpionidae AND poison	4 March 2026	0
6	Scorpionidae AND toxicant	4 March 2026	0
7	Scorpionidae AND zootoxin	4 March 2026	0
8	taxonomy_id 6855 = Scorpiones	4 March 2026	7578
9	arachnid	4 March 2026	231
10	Invertebrate AND toxin	4 March 2026	2086
11	SCORPION	4 March 2026	19294
12	venom	5 March 2026	193702
13	toxin AND Arthropoda	5 March 2026	33,583
14	(taxonomy_id:6865) buthus species	5 March 2026	353

Table S2. Protein identifications by Proteome Discoverer 3.2 against the UniProtKB/Swiss-Prot database.

Accession	Description	Coverage [%]	# Peptides	# PSMs	# Unique Peptides	# AAs	MW [kDa]	calc. pI	Sequest HT Score	# Peptides (Sequest HT)	# Protein Groups
P84744	Potassium channel toxin alpha-KTx 9.5 OS=*Buthus occitanus tunetanus* OX=6871 PE=1 SV=1	93	2	18	2	28	2.9	5.62	36.51	2	1
B8XH07	Putative depressant toxin Tx131 (Fragment) OS=*Buthus israelis* OX=2899555 PE=2 SV=1	66	1	2	1	38	4.1	5.02	4.29	1	1
P55903	Beta-insect depressant toxin BotIT4 OS=*Buthus occitanus tunetanus* OX=6871 PE=1 SV=1	66	3	4	3	61	6.8	5.31	11.69	3	1
Q17254	Alpha-insect toxin Bot14 OS=*Buthus occitanus tunetanus* OX=6871 PE=2 SV=1	59	5	13	5	85	9.2	8.5	35.53	5	1
B8XGZ0	Putative alpha toxin Tx613 OS=*Buthus israelis* OX=2899555 PE=2 SV=1	52	4	12	4	85	9.5	7.53	31.09	4	1
P56215	Potassium channel toxin alpha-KTx 8.1 OS=*Androctonus mauritanicus mauritanicus* OX=6860 PE=1 SV=1	48	1	4	1	29	3.2	4.72	11.55	1	1
B8XGZ8	Lipolysis-activating peptide 1-beta chain OS=*Buthus israelis* OX=2899555 PE=3 SV=1	47	9	78	9	98	11.2	7.93	152.26	9	1
P84646	Alpha-toxin OD1 OS=*Odontobuthus doriae* OX=342590 PE=1 SV=1	46	3	9	1	65	7.2	7.5	15.91	3	1
P01486	Alpha-toxin Bot11 OS=*Buthus occitanus tunetanus* OX=6871 PE=1 SV=1	46	4	16	3	65	7.5	7.87	22.24	4	1
B8XH01	Lipolysis-activating peptide 1-alpha chain OS=*Buthus israelis* OX=2899555 PE=2 SV=1	44	4	34	4	98	11.2	8.18	54.75	4	1
P59863	Beta-toxin BotIT2 OS=*Buthus occitanus tunetanus* OX=6871 PE=1 SV=1	43	3	5	3	60	6.9	4.84	15.97	3	1
B8XGY5	Putative alpha toxin Tx222 OS=*Buthus israelis* OX=2899555 PE=2 SV=1	42	3	11	2	85	9.5	8.1	13.61	3	1
P0DJH8	Alpha-toxin Bu1 OS=*Buthacus macrocentrus* OX=1143368 PE=1 SV=1	39	4	14	3	67	7.5	8.48	26.12	4	1
B8XGY7	Putative alpha toxin Tx405 OS=*Buthus israelis* OX=2899555 PE=2 SV=1	36	3	10	2	85	9.3	7.5	24.52	3	1
B8XGX9	Putative alpha toxin Tx2 OS=*Buthus israelis* OX=2899555 PE=2 SV=1	34	2	6	1	87	9.6	7.5	10.88	2	1
A0A059UD62	Phospholipase A2 (Fragment) OS=*Mesobuthus gibbosus* OX=123226 PE=2 SV=1	31	5	15	3	128	15.2	8.32	23.58	5	1
P59896	Toxin BomPI OS=*Buthus occitanus mardochei* OX=6869 PE=1 SV=1	27	1	2	1	66	7.4	7.55	5.61	1	1
B8XH04	Putative excitatory toxin Tx402 OS=*Buthus israelis* OX=2899555 PE=2 SV=1	27	3	13	3	88	10.2	7.85	8.55	3	1
B8XGZ2	Putative alpha toxin Tx644 OS=*Buthus israelis* OX=2899555 PE=2 SV=1	27	2	4	2	81	8.8	7.88	6.36	2	1
B8XH19	Putative depressant toxin Tx695 OS=*Buthus israelis* OX=2899555 PE=2 SV=1	27	1	1	1	81	9.1	8.65	2.87	1	1
B8XGY4	Putative alpha toxin Tx68 OS=*Buthus israelis* OX=2899555 PE=2 SV=1	27	2	3	2	85	9.6	7.56	3.42	2	1
C0HM60	Toxin Bot33 OS=*Buthus occitanus tunetanus* OX=6871 PE=1 SV=2	26	1	4	1	38	4	8.72	1.67	1	1
C0HJQ2	Potassium channel toxin alpha-KTx 19.2 OS=*Buthus occitanus tunetanus* OX=6871 PE=1 SV=1	26	1	3	1	31	3.4	8.73	3.45	1	1
A0A088D9U2	Potassium channel blocker pMeKTx28-2 OS=*Mesobuthus eupeus* OX=34648 PE=2 SV=1	26	2	6	2	90	10.3	8.65	10.75	2	1
A0A5P8U2M3	Toxin (Fragment) OS=*Mesobuthus eupeus* OX=34648 PE=2 SV=1	25	1	3	1	68	7.5	8.31	3.81	1	1
A0A0K0LBY9	Calcium channel blocker AbCaTx1 OS=*Androctonus bicolor* OX=748906 PE=2 SV=1	25	1	1	1	64	7.1	6.71	0	1	1
B8XH47	Potassium channel toxin alpha-KTx Tx790 OS=*Buthus israelis* OX=2899555 PE=2 SV=1	24	1	2	1	62	7.2	6.39	3.71	1	1
A0A0K0LBV3	Sodium channel blocker AbNaTx25 OS=*Androctonus bicolor* OX=748906 PE=2 SV=1	23	2	4	2	86	10	8.76	4.15	2	1
B8XH30	Potassium channel toxin alpha-KTx Tx308 OS=*Buthus israelis* OX=2899555 PE=2 SV=1	21	1	2	1	62	7.4	8.4	2.26	1	1
B8XH40	Potassium channel toxin BuTXK-beta OS=*Buthus israelis* OX=2899555 PE=3 SV=1	21	2	5	2	91	10.2	8.57	6.38	2	1
A0A0U4GT75	Sodium channel toxin NaTx8 OS=*Odontobuthus doriae* OX=342590 GN=NaTx8 PE=2 SV=1	20	2	32	2	98	11	7.61	57.14	2	1
A0A7T9L322	Hyaluronidase OS=*Androctonus crassicauda* OX=122909 GN=Hyal PE=2 SV=1	20	7	27	6	410	47.5	8.37	42.14	7	1
A0A0K0LC76	Cellular protein AbCp-44 (Fragment) OS=*Androctonus bicolor* OX=748906 PE=2 SV=1	20	2	6	2	99	11.3	6.9	3.56	2	1
P01488	Alpha-toxin Bot1 OS=*Buthus occitanus tunetanus* OX=6871 PE=1 SV=2	20	1	1	1	65	7.3	6.92	0	1	1

Accession	Description	Coverage [%]	# Peptides	# PSMs	# Unique Peptides	# AAs	MW [kDa]	calc. pI	Sequest HT Score	# Peptides (Sequest HT)	# Protein Groups
B8XGY3	Putative alpha toxin Tx120 OS=*Buthus israelis* OX=2899555 PE=2 SV=1	20	2	6	1	86	9.3	8.48	5.8	2	1
P15224	Toxin Os1 OS=*Orthochirus scrobiculosus* OX=6892 PE=1 SV=1	20	1	1	1	66	7.6	7.88	0	1	1
P45660	Potassium channel toxin alpha-KTx 16.5 OS=*Leiurus hebraeus* OX=2899558 PE=1 SV=1	19	1	2	1	36	4.1	8.75	3.66	1	1
P84809	Lipolysis-activating peptide 1-beta chain OS=*Buthus occitanus tunetanus* OX=6871 PE=1 SV=1	19	2	7	2	95	10.4	5.95	7.42	2	1
C5J8B0	Hemocyanin-like peptide (Fragment) OS=*Opisthacanthus cayaporum* OX=573324 GN=HC PE=2 SV=1	18	2	4	1	179	20.5	5.3	7.18	2	1
B8XH08	Putative depressant toxin Tx233 OS=*Buthus israelis* OX=2899555 PE=2 SV=1	18	1	1	1	85	9.4	7.87	2.47	1	1
B8XH09	Putative depressant toxin Tx273 OS=*Buthus israelis* OX=2899555 PE=2 SV=1	17	2	7	2	86	9.7	8.76	6.56	2	1
F8THJ4	CRISP3 (Fragment) OS=*Hottentotta judaicus* OX=6863 PE=2 SV=1	16	3	13	3	192	21.2	8.78	26.64	3	1
P86100	Hyaluronidase-1 OS=*Olivierus martensii* OX=34649 PE=1 SV=2	15	5	9	4	410	47.4	8.16	22.48	5	1
A0A1W7R9R4	Ubiquitin-ribosomal protein eS31 fusion protein OS=*Hadrurus spadix* OX=141984 PE=3 SV=1	14	2	2	2	156	18	9.72	2.61	2	1
A0A2I9LP14	Hemocyanin subunit F OS=*Centruroides hentzi* OX=88313 PE=3 SV=1	14	8	19	4	626	71.9	5.96	20.9	8	1
B8XH20	Putative depressant toxin Tx700 OS=*Buthus israelis* OX=2899555 PE=2 SV=1	14	1	1	1	80	8.9	7.87	0	1	1
F1CIX4	U1-buthitoxin-Hj1a OS=*Hottentotta judaicus* OX=6863 PE=2 SV=1	13	1	1	1	75	8.7	8.02	0	1	1
A0A1W7RB46	Actin OS=*Hadrurus spadix* OX=141984 PE=3 SV=1	13	3	5	3	376	41.8	5.48	4.2	3	1
P58296	Toxin Cn11 OS=*Centruroides noxius* OX=6878 PE=1 SV=1	13	1	2	1	63	7	6.93	1.66	1	1
E7CZZ1	Sodium channel neurotoxin MeuNaTxalpha-12 (Fragment) OS=*Mesobuthus eupeus* OX=34648 PE=2 SV=1	13	1	4	1	71	7.8	7.88	6.18	1	1
B8XH12	Putative depressant toxin Tx438 OS=*Buthus israelis* OX=2899555 PE=2 SV=1	13	1	1	1	80	8.8	8.1	1.97	1	1
A0AA50AAI4	Phospholipase A2 OS=*Androctonus crassicauda* OX=122909 PE=2 SV=1	12	4	14	2	213	25	6.95	23.72	4	1
F1CJ13	protein disulfide-isomerase (Fragment) OS=*Hottentotta judaicus* OX=6863 PE=2 SV=1	11	4	5	4	319	37.2	5.33	3.54	4	1
P80476	Hemocyanin AA6 chain OS=*Androctonus australis* OX=6858 PE=1 SV=1	11	6	10	2	626	71.7	5.82	13.18	6	1
B8XH44	Potassium channel toxin alpha-KTx 27.1 OS=*Buthus israelis* OX=2899555 PE=3 SV=1	11	1	1	1	73	8.2	8.48	0	1	1
F1CJ49	U5-buthitoxin-Hj1a OS=*Hottentotta judaicus* OX=6863 PE=2 SV=1	10	1	2	1	87	9.7	7.5	1.93	1	1
A0A0U4H7F5	Potassium channel toxin KTx11 OS=*Odontobuthus doriae* OX=342590 GN=KTx11 PE=2 SV=1	10	1	1	1	91	10.2	8.57	1.6	1	1
A0A0U1SF04	Peptidase M14 domain-containing protein (Fragment) OS=*Isometrus maculatus* OX=497827 PE=2 SV=1	9	2	6	2	201	23.1	5.14	12.63	2	1
C6H100	Hemocyanin subunit 5a OS=*Pandinus imperator* OX=55084 GN=hc5a PE=2 SV=1	9	6	8	3	627	71.9	6.44	10.22	6	1
Q0G8J8	High-affinity serotonin transporter protein (Fragment) OS=*Opistophthalmus* sp. CD-2006 OX=400501 GN=SERT PE=2 SV=1	9	1	1	1	174	19.9	8.15	2.13	1	1
A0A0U1S617	LCN-type CS-alpha/beta domain-containing protein OS=*Isometrus maculatus* OX=497827 PE=2 SV=1	9	1	1	1	92	10.5	7.93	1.99	1	1
A0A146CJ36	Venom peptide meuPep28 OS=*Mesobuthus eupeus* OX=34648 PE=2 SV=1	9	1	1	1	94	10.5	8.1	0	1	1
B8XH17	Putative depressant toxin Tx580 OS=*Buthus israelis* OX=2899555 PE=2 SV=1	8	1	4	1	83	9.5	8.46	3.95	1	1
P59865	Toxin Cll7 OS=*Centruroides limpidus* OX=6876 PE=2 SV=1	8	1	2	1	85	9.6	7.81	0	1	1
A0A2I9LNZ2	Endoplasmic reticulum resident protein 44 OS=*Centruroides hentzi* OX=88313 PE=4 SV=1	8	3	4	3	418	48.3	6.35	7.62	3	1
A0A1E1WWC5	Putative hemocyanin subunit OS=*Tityus obscurus* OX=1221240 PE=3 SV=1	8	5	10	4	624	71.5	6.19	9.08	5	1
A0A2I9LNW8	Protein D3 OS=*Centruroides hentzi* OX=88313 PE=4 SV=1	8	1	2	1	180	20.3	6.1	5.38	1	1
A0A1W7RAF1	Histone H4 OS=*Hadrurus spadix* OX=141984 PE=3 SV=1	8	1	4	1	103	11.4	11.36	0	1	1
A0A1W7RAY3	Tubulin alpha chain OS=*Hadrurus spadix* OX=141984 PE=3 SV=1	8	1	1	1	451	50.1	5.06	4.05	1	1

Accession	Description	Coverage [%]	# Peptides	# PSMs	# Unique Peptides	# AAs	MW [kDa]	calc. pI	Sequest HT Score	# Peptides (Sequest HT)	# Protein Groups
A0A0U4QF63	Sodium channel toxin NaTx7 OS=*Odontobuthus doriae* OX=342590 GN=NaTx7 PE=2 SV=1	7	1	41	1	97	10.9	8.54	47.49	1	1
A0A2I9LNZ4	Fatty acid-binding protein OS=*Centruroides hentzi* OX=88313 PE=3 SV=1	7	1	1	1	131	14.8	5.43	0	1	1
A0A1E1WVS7	Putative hemocyanin subunit OS=*Tityus obscurus* OX=1221240 PE=3 SV=1	7	4	8	3	632	72.7	5.83	13.78	4	1
A0A2I9LP22	Hemocyanin AA6 chain OS=*Centruroides hentzi* OX=88313 PE=3 SV=1	7	5	8	1	627	72	5.77	10.91	5	1
A0A1E1WVN1	Putative hemocyanin subunit OS=*Tityus obscurus* OX=1221240 PE=4 SV=1	6	4	10	3	628	72.2	6.55	5.18	4	1
A0A0U4HEU8	Venom protein VP4 OS=*Odontobuthus doriae* OX=342590 GN=VP4 PE=2 SV=1	5	1	1	1	143	16	7.24	0	1	1
A0A2I9LPD2	Mesencephalic astrocyte-derived neurotrophic factor homolog OS=*Centruroides hentzi* OX=88313 PE=3 SV=1	5	1	3	1	173	19.9	6.96	0	1	1
A0A2I9LPS4	limulus clotting factor C OS=*Centruroides hentzi* OX=88313 PE=4 SV=1	5	1	2	1	309	34.6	6	4.14	1	1
F1CJ43	Putative alpha-2-macroglobulin (Fragment) OS=*Hottentotta judaicus* OX=6863 PE=2 SV=1	4	1	2	1	179	20.4	8.13	0	1	1
A0A2I9LP74	peptidylglycine monooxygenase OS=*Centruroides hentzi* OX=88313 PE=3 SV=1	4	2	5	2	347	39	7.23	7.92	2	1
A0A1E1WVL6	Putative hemocyanin subunit OS=*Tityus obscurus* OX=1221240 PE=4 SV=1	4	3	5	2	637	73.2	6.71	6.31	3	1
A0A2I9LP44	Insulin-like growth factor-binding protein OS=*Centruroides hentzi* OX=88313 PE=4 SV=1	3	1	1	1	254	28.5	6.8	0	1	1
A0A1E1WVR2	Putative transferrin OS=*Tityus obscurus* OX=1221240 PE=3 SV=1	3	2	5	2	711	78.7	5.3	7.29	2	1
A0A1W7R9Z2	Protein disulfide-isomerase OS=*Hadrurus spadix* OX=141984 PE=3 SV=1	2	1	4	1	491	56.4	5.11	10.91	1	1
A0A1S5QN46	Carboxypeptidase E OS=*Tityus serrulatus* OX=6887 PE=2 SV=1	2	1	1	1	472	53.8	5.58	2.05	1	1
A0A2I9LPT3	vesicle-fusing ATPase OS=*Centruroides hentzi* OX=88313 PE=4 SV=1	2	1	1	1	806	89.3	5.6	0	1	1
A0A1W7RAN3	Elongation factor 1-alpha OS=*Hadrurus spadix* OX=141984 PE=3 SV=1	2	1	1	1	464	51.2	9.01	0	1	1
A0A2I9LNV7	Cysteine-rich secretory protein OS=*Centruroides hentzi* OX=88313 PE=4 SV=1	2	1	6	1	418	47.3	6.81	7.88	1	1
T1DPC1	CAP-Lyc-1 OS=*Lychas buchari* OX=1330406 PE=2 SV=1	2	1	3	1	422	47	7.53	5.7	1	1
A0A2I9LNS6	Acid phosphatase OS=*Centruroides hentzi* OX=88313 PE=3 SV=1	2	1	1	1	379	43.2	5.12	0	1	1
U6BLN5	Venom metalloproteinase BumaMPs1 OS=*Olivierus martensii* OX=34649 PE=2 SV=1	2	1	2	1	393	44.4	5.64	1.74	1	1
A0A1S5QN38	Aminopeptidase OS=*Tityus serrulatus* OX=6887 PE=2 SV=1	2	1	2	1	961	111.2	6.54	2.26	1	1
A0A1W7RAC2	Heat shock 70 kDa protein cognate 3 OS=*Hadrurus spadix* OX=141984 PE=3 SV=1	2	1	3	1	657	72.9	5.34	6.39	1	1
A0A1W7RAG2	Gamma-glutamyltranspeptidase 1 OS=*Hadrurus spadix* OX=141984 PE=4 SV=1	1	1	1	1	622	69.1	6.96	0	1	1
A0A2I9LP76	Myosin heavy chain, muscle-like protein OS=*Centruroides hentzi* OX=88313 PE=3 SV=1	1	2	2	2	1955	224.4	6.02	2.51	2	**1**

Column definitions — # Peptides: distinct peptide sequences matched to the protein; # Unique Peptides: peptides assigned uniquely to a single protein group (proteotypic); # PSMs: peptide-spectrum matches; Sequest HT Score: cumulative Sequest HT identification score for the protein; # Peptides (Sequest HT): peptides identified by the Sequest HT search engine; # Protein Groups: number of protein groups sharing the matched peptides.

Protein descriptions are reproduced verbatim from UniProtKB (organism name OS and taxon identifier OX as annotated by UniProt). Where the currently accepted taxonomic name differs from the UniProt annotation, it is indicated separately — e.g., UniProt *Buthus occitanus mardochei* (OX = 6869) corresponds to the currently valid species *Buthus mardochei* Simon, 1878.

Table S3. Protein identifications by Proteome Discoverer 3.2 against the UniProtKB/Swiss-Prot database after BLAST-guided alignment expansion.

Bold accession numbers denote proteins newly identified at this analytical stage (not present in Table S2).

Accession	Description	Coverage [%]	# Peptides	# PSMs	# Unique Peptides	# AAs	MW [kDa]	calc. pI	Sequest HT Score	# Peptides (Sequest HT)	# Protein Groups	Modifications
P84744	Potassium channel toxin alpha-KTx 9.5 OS=*Buthus occitanus tunetanus* OX=6871 PE=1 SV=1	93	2	28	2	28	2.9	5.62	40.07	2	1	
**P60257**	Toxin Boma6c OS=*Buthus occitanus mardochei* OX=6869 PE=3 SV=1	76	5	16	5	66	7.4	8.31	26.46	5	1	Oxidation [C12]
Q17254	Alpha-insect toxin Bot14 OS=*Buthus occitanus tunetanus* OX=6871 PE=2 SV=1	59	5	19	5	85	9.2	8.5	38.56	5	1	
B8XGZ8	Lipolysis-activating peptide 1-beta chain OS=*Buthus israelis* OX=2899555 PE=3 SV=1	51	12	185	12	98	11.2	7.93	240.19	12	1	
P56215	Potassium channel toxin alpha-KTx 8.1 OS=*Androctonus mauritanicus mauritanicus* OX=6860 PE=1 SV=1	48	1	5	1	29	3.2	4.72	10.92	1	1	
P84646	Alpha-toxin OD1 OS=*Odontobuthus doriae* OX=342590 PE=1 SV=1	46	3	12	2	65	7.2	7.5	21.8	3	1	
P01486	Alpha-toxin Bot11 OS=*Buthus occitanus tunetanus* OX=6871 PE=1 SV=1	46	4	16	3	65	7.5	7.87	22.11	4	1	
B8XH01	Lipolysis-activating peptide 1-alpha chain OS=*Buthus israelis* OX=2899555 PE=2 SV=1	44	4	41	4	98	11.2	8.18	61.32	4	1	
P59863	Beta-toxin BotIT2 OS=*Buthus occitanus tunetanus* OX=6871 PE=1 SV=1	43	3	8	3	60	6.9	4.84	18.65	3	1	
P0DJH8	Alpha-toxin Bu1 OS=*Buthacus macrocentrus* OX=1143368 PE=1 SV=1	39	4	22	3	67	7.5	8.48	33.81	4	1	
P01488	Alpha-toxin Bot1 OS=*Buthus occitanus tunetanus* OX=6871 PE=1 SV=2	34	2	3	2	65	7.3	6.92	0	2	1	
**D5HR51**	Alpha-toxin Ac2 OS=*Androctonus crassicauda* OX=122909 PE=3 SV=1	33	2	9	1	85	9.5	8.29	11.75	2	1	
**P45697**	Alpha-like toxin BmK M1 OS=*Olivierus martensii* OX=34649 PE=1 SV=2	32	2	7	2	84	9.5	7.88	0	2	1	
P80476	Hemocyanin AA6 chain OS=*Androctonus australis* OX=6858 PE=1 SV=1	30	16	65	5	626	71.7	5.82	31.97	16	1	
**P83407**	Potassium channel toxin alpha-KTx 19.1 OS=*Olivierus martensii* OX=34649 PE=1 SV=1	29	1	1	1	31	3.3	8.73	0	1	1	Oxidation [C3]
**P68721**	Beta-insect excitatory toxin LqhIT1a OS=*Leiurus hebraeus* OX=2899558 PE=3 SV=1	27	2	2	2	88	9.9	8.09	0	2	1	
P59896	Toxin BomPI OS=*Buthus occitanus mardochei* OX=6869 PE=1 SV=1	27	1	3	1	66	7.4	7.55	5.74	1	1	
**Q56TT9**	Alpha-insect toxin BjaIT OS=*Hottentotta judaicus* OX=6863 PE=1 SV=1	27	2	6	2	85	9.3	7.81	16.39	2	1	
**B8XH46**	Potassium channel toxin alpha-KTx 9.8 OS=*Buthus israelis* OX=2899555 PE=3 SV=1	27	1	1	1	56	6	5.1	1.76	1	1	
A0A7T9L322	Hyaluronidase OS=*Androctonus crassicauda* OX=122909 GN=Hyal PE=2 SV=1	27	12	51	12	410	47.5	8.37	48.25	12	1	
**Q2YHM1**	Neurotoxin 8-related gene product 1/2/3 OS=*Androctonus mauritanicus mauritanicus* OX=6860 GN=NTVIIIrgp1 PE=3 SV=1	26	1	1	1	86	9.6	8.1	0	1	1	
**P86399**	Neurotoxin lambda-MeuTx OS=*Mesobuthus eupeus* OX=34648 PE=1 SV=2	25	1	1	1	64	7.2	7.12	0	1	1	
P55903	Beta-insect depressant toxin BotIT4 OS=*Buthus occitanus tunetanus* OX=6871 PE=1 SV=1	25	2	4	2	61	6.8	5.31	11.77	2	1	
**P59864**	Beta-insect depressant toxin BotIT6 OS=*Buthus occitanus tunetanus* OX=6871 PE=1 SV=1	24	2	8	2	62	7.3	8.1	8.46	2	1	
**Q9UAC9**	Beta-toxin BmKAS OS=*Olivierus martensii* OX=34649 PE=1 SV=1	24	2	5	2	85	9.8	8.62	4.19	2	1	
A0A2I9LP14	Hemocyanin subunit F OS=*Centruroides hentzi* OX=88313 PE=3 SV=1	23	15	38	9	626	71.9	5.96	25.49	15	1	

Accession	Description	Coverage [%]	# Peptides	# PSMs	# Unique Peptides	# AAs	MW [kDa]	calc. pI	Sequest HT Score	# Peptides (Sequest HT)	# Protein Groups	Modifications
B8XH30	Potassium channel toxin alpha-KTx Tx308 OS=*Buthus israelis* OX=2899555 PE=2 SV=1	21	1	2	1	62	7.4	8.4	1.62	1	1	
B8XH40	Potassium channel toxin BuTXK-beta OS=*Buthus israelis* OX=2899555 PE=3 SV=1	21	2	6	2	91	10.2	8.57	7.03	2	1	
**Q8I0L5**	Potassium channel toxin alpha-KTx 15.2 OS=*Olivierus martensii* OX=34649 PE=1 SV=1	20	1	2	1	59	6.2	8.69	0	1	1	
**Q9BLM4**	Toxin AahP1005 OS=*Androctonus australis* OX=6858 PE=2 SV=1	19	2	10	2	85	9.5	8.46	5.93	2	1	
A0A2I9LP22	Hemocyanin AA6 chain OS=*Centruroides hentzi* OX=88313 PE=3 SV=1	17	12	58	1	627	72	5.77	29.37	12	1	
A0AA50AAI4	Phospholipase A2 OS=*Androctonus crassicauda* OX=122909 PE=2 SV=1	16	6	28	6	213	25	6.95	32.17	6	1	
**Q9NJC6**	Potassium channel toxin BmTXK-beta OS=*Olivierus martensii* OX=34649 PE=3 SV=1	16	1	8	1	90	10.4	8.82	7.82	1	1	
E7CZZ1	Sodium channel neurotoxin MeuNaTxalpha-12 (Fragment) OS=*Mesobuthus eupeus* OX=34648 PE=2 SV=1	13	1	4	1	71	7.8	7.88	6.2	1	1	
**B8XH42**	Potassium channel toxin alpha-KTx 16.6 OS=*Buthus israelis* OX=2899555 PE=2 SV=1	12	1	1	1	58	6.5	8.12	0	1	1	
**P86406**	MeuNaTxbeta-1 OS=*Mesobuthus eupeus* OX=34648 PE=1 SV=2	12	1	1	1	86	10	8.29	0	1	1	
**P0C292**	Toxin Acra1 OS=*Androctonus crassicauda* OX=122909 PE=1 SV=1	11	1	4	1	80	8.9	7.14	2.9	1	1	
B8XH44	Potassium channel toxin alpha-KTx 27.1 OS=*Buthus israelis* OX=2899555 PE=3 SV=1	11	1	2	1	73	8.2	8.48	0	1	1	
C6H100	Hemocyanin subunit 5a OS=*Pandinus imperator* OX=55084 GN=hc5a PE=2 SV=1	10	8	45	3	627	71.9	6.44	36.56	8	1	
**P69939**	Potassium channel toxin AaTXK-beta OS=*Androctonus australis* OX=6858 PE=1 SV=1	10	1	3	1	91	10.1	8.76	1.62	1	1	
**D9U2A4**	Lipolysis-activating peptide 1-alpha chain OS=*Lychas mucronatus* OX=172552 PE=3 SV=1	9	1	1	1	97	11.1	7.59	3.22	1	1	
A0A1E1WVL6	Putative hemocyanin subunit OS=*Tityus obscurus* OX=1221240 PE=4 SV=1	8	6	38	4	637	73.2	6.71	23.24	6	1	
A0A0U4QF63	Sodium channel toxin NaTx7 OS=*Odontobuthus doriae* OX=342590 GN=NaTx7 PE=2 SV=1	7	1	88	1	97	10.9	8.54	86.68	1	1	
**O77091**	Beta-insect excitatory toxin BmK IT-AP OS=*Olivierus martensii* OX=34649 PE=1 SV=1	7	1	12	1	90	10.2	5.36	1.64	1	1	
**A0A367L118**	Heat-labile enterotoxin OS=*Ophiocordyceps polyrhachis-furcata* BCC 54312 OX=1330021 GN=L249_6316 PE=4 SV=1	3	1	2	1	375	42	4.87	0	1	1	

Column definitions — # Peptides: distinct peptide sequences matched to the protein; # Unique Peptides: peptides assigned uniquely to a single protein group (proteotypic); # PSMs: peptide-spectrum matches; Sequest HT Score: cumulative Sequest HT identification score for the protein; # Peptides (Sequest HT): peptides identified by the Sequest HT search engine; # Protein Groups: number of protein groups sharing the matched peptides; Modifications: PTM sites localised at a Proteome Discoverer site probability ≥ 0.75.

Table S4. Protein identifications by Proteome Discoverer 3.2 against the UniProtKB/Swiss-Prot database, including sequence variants.

Accession	Description	Coverage [%]	# Peptides	# PSMs	# Unique Peptides	# AAs	MW [kDa]	calc. pI	Sequest HT Score	# Peptides (Sequest HT)	# Protein Groups	Modifications
P84744	Potassium channel toxin alpha-KTx 9.5 OS=*Buthus occitanus tunetanus* OX=6871 PE=1 SV=1	93	2	30	2	28	2.9	5.62	35.28	2	1	
P60257_V	Toxin Boma6c_V OS=*Buthus occitanus mardochei*_V OX=6869 PE=3 SV=1	67	5	16	5	66	7.5	8.31	25.61	5	1	
P84646_V2	Alpha-toxin OD1_v2 OS=*Odontobuthus doriae*_V2 OX=342590 PE=1 SV=1	58	5	11	4	65	7.3	7.87	14.88	5	1	
Q17254_v	Alpha-insect toxin Bot14_V OS=*Buthus occitanus tunetanus*_v OX=6871 PE=2 SV=1	56	5	17	5	89	9.6	8.66	39.47	5	1	
P56215	Potassium channel toxin alpha-KTx 8.1 OS=*Androctonus mauritanicus mauritanicus* OX=6860 PE=1 SV=1	48	1	5	1	29	3.2	4.72	11.28	1	1	
P01488_V	Alpha-toxin Bot1_V OS=*Buthus occitanus tunetanus*_V OX=6871 PE=1 SV=2	48	2	5	2	65	7.3	7.55	3.74	2	1	
P01486	Alpha-toxin Bot11 OS=*Buthus occitanus tunetanus* OX=6871 PE=1 SV=1	46	4	16	3	65	7.5	7.87	20.05	4	1	
Q9UAC9_v2	Beta-toxin BmKAS_v2 OS=*Olivierus martensii*_v2 OX=34649 PE=1 SV=1	41	3	7	3	85	9.7	8.46	6.32	3	1	
P0DJH8_v	Alpha-toxin Bu1_V OS=*Buthacus macrocentrus*_v OX=1143368 PE=1 SV=1	39	4	18	3	67	7.5	8.48	24.22	4	1	
D5HR51	Alpha-toxin Ac2 OS=*Androctonus crassicauda* OX=122909 PE=3 SV=1	33	2	8	1	85	9.5	8.29	8.34	2	1	
B8XH01	Lipolysis-activating peptide 1-alpha chain OS=*Buthus israelis* OX=2899555 PE=2 SV=1	31	3	38	3	98	11.2	8.18	56.74	3	1	
P83407	Potassium channel toxin alpha-KTx 19.1 OS=*Olivierus martensii* OX=34649 PE=1 SV=1	29	1	1	1	31	3.3	8.73	0	1	1	Oxidation [C3]
A0A0U4QF63_v2	Sodium channel toxin NaTx7_v2 OS=*Odontobuthus doriae* OX=342590 GN=NaTx7 PE=2 SV=1	28	3	153	3	97	10.9	8.69	146.29	3	1	
Q56TT9	Alpha-insect toxin BjaIT OS=*Hottentotta judaicus* OX=6863 PE=1 SV=1	27	2	5	2	85	9.3	7.81	14.9	2	1	
B8XH46	Potassium channel toxin alpha-KTx 9.8 OS=*Buthus israelis* OX=2899555 PE=3 SV=1	27	1	1	1	56	6	5.1	0	1	1	
Q2YHM1	Neurotoxin 8-related gene product 1/2/3 OS=*Androctonus mauritanicus mauritanicus* OX=6860 GN=NTVIIIrgp1 PE=3 SV=1	26	1	1	1	86	9.6	8.1	0	1	1	
Q9NJC6_v	Potassium channel toxin BmTXK-beta_v OS=*Olivierus martensii*_vOX=34649 PE=3 SV=1	26	2	11	2	90	10.5	8.65	12.95	2	1	
P86399	Neurotoxin lambda-MeuTx OS=*Mesobuthus eupeus* OX=34648 PE=1 SV=2	25	1	1	1	64	7.2	7.12	0	1	1	
P59863	Beta-toxin BotIT2 OS=*Buthus occitanus tunetanus* OX=6871 PE=1 SV=1	25	2	5	2	60	6.9	4.84	12.44	2	1	
P55903	Beta-insect depressant toxin BotIT4 OS=*Buthus occitanus tunetanus* OX=6871 PE=1 SV=1	25	1	2	1	61	6.8	5.31	9.6	1	1	
P59864	Beta-insect depressant toxin BotIT6 OS=*Buthus occitanus tunetanus* OX=6871 PE=1 SV=1	24	2	10	2	62	7.3	8.1	6.71	2	1	
A0A7T9L322	Hyaluronidase OS=*Androctonus crassicauda* OX=122909 GN=Hyal PE=2 SV=1	23	9	48	9	410	47.5	8.37	39.73	9	1	
B8XH30	Potassium channel toxin alpha-KTx Tx308 OS=*Buthus israelis* OX=2899555 PE=2 SV=1	21	1	2	1	62	7.4	8.4	2.29	1	1	
B8XH40	Potassium channel toxin BuTXK-beta OS=*Buthus israelis* OX=2899555 PE=3 SV=1	21	2	5	2	91	10.2	8.57	6.55	2	1	
Q9BLM4	Toxin AahP1005 OS=*Androctonus australis* OX=6858 PE=2 SV=1	19	2	9	2	85	9.5	8.46	5.86	2	1	

Accession	Description	Coverage [%]	# Peptides	# PSMs	# Unique Peptides	# AAs	MW [kDa]	calc. pI	Sequest HT Score	# Peptides (Sequest HT)	# Protein Groups	Modifications
P68721	Beta-insect excitatory toxin LqhIT1a OS=*Leiurus hebraeus* OX=2899558 PE=3 SV=1	18	1	1	1	88	9.9	8.09	0	1	1	
A0A2I9LP14	Hemocyanin subunit F OS=*Centruroides hentzi* OX=88313 PE=3 SV=1	16	10	32	5	626	71.9	5.96	22.93	10	1	
A0AA50AAI4	Phospholipase A2 OS=*Androctonus crassicauda* OX=122909 PE=2 SV=1	15	5	25	5	213	25	6.95	27.68	5	1	
P80476	Hemocyanin AA6 chain OS=*Androctonus australis* OX=6858 PE=1 SV=1	13	7	46	3	626	71.7	5.82	29.3	7	1	
E7CZZ1	Sodium channel neurotoxin MeuNaTxalpha-12 (Fragment) OS=*Mesobuthus eupeus* OX=34648 PE=2 SV=1	13	1	3	1	71	7.8	7.88	6.22	1	1	
P0C292	Toxin Acra1 OS=*Androctonus crassicauda* OX=122909 PE=1 SV=1	11	1	4	1	80	8.9	7.14	2.84	1	1	
B8XH44	Potassium channel toxin alpha-KTx 27.1 OS=*Buthus israelis* OX=2899555 PE=3 SV=1	11	1	2	1	73	8.2	8.48	0	1	1	
P86406	MeuNaTxbeta-1 OS=*Mesobuthus eupeus* OX=34648 PE=1 SV=2	10	1	20	1	124	14.4	8.12	23.99	1	1	
P69939	Potassium channel toxin AaTXK-beta OS=*Androctonus australis* OX=6858 PE=1 SV=1	10	1	3	1	91	10.1	8.76	1.62	1	1	
D9U2A4	Lipolysis-activating peptide 1-alpha chain OS=*Lychas mucronatus* OX=172552 PE=3 SV=1	9	1	1	1	97	11.1	7.59	3.22	1	1	
C6H100	Hemocyanin subunit 5a OS=*Pandinus imperator* OX=55084 GN=hc5a PE=2 SV=1	9	6	45	2	627	71.9	6.44	34.75	6	1	
O77091	Beta-insect excitatory toxin BmK IT-AP OS=*Olivierus martensii* OX=34649 PE=1 SV=1	7	1	14	1	90	10.2	5.36	1.64	1	1	
A0A2I9LP22	Hemocyanin AA6 chain OS=*Centruroides hentzi* OX=88313 PE=3 SV=1	7	5	45	1	627	72	5.77	26.85	5	1	
A0A1E1WVL6	Putative hemocyanin subunit OS=*Tityus obscurus* OX=1221240 PE=4 SV=1	6	5	37	3	637	73.2	6.71	21.35	5	1	

Column definitions — # Peptides: distinct peptide sequences matched to the protein; # Unique Peptides: peptides assigned uniquely to a single protein group (proteotypic); # PSMs: peptide-spectrum matches; Sequest HT Score: cumulative Sequest HT identification score for the protein; # Peptides (Sequest HT): peptides identified by the Sequest HT search engine; # Protein Groups: number of protein groups sharing the matched peptides; Modifications: PTM sites localised at a Proteome Discoverer site probability ≥ 0.75.

Table S5. Protein identifications by Proteome Discoverer 3.2 against the UniProtKB/Swiss-Prot database, combining sequence variants, deleted signal-peptide/propeptide regions, BLAST-derived homologs and post-translational modifications (PTMs). Counts are reported per entry; the dataset-level totals given in the main text (Section 2.2) are deduplicated across the run and therefore differ from the arithmetic sums of these columns.

Accession	Description	BLAST best-hit organism	Functional category	Confidence tier	Coverage [%]	# Peptides	# Unique Peptides	# PSMs	MW [kDa]	calc. pI	Modifications	Comment/validation note
P60257_V+V2_F>A17	Toxin Boma6c_V OS=*Buthus occitanus mardochei_V* OX=6869 PE=3 SV=1	*Buthus mardochei*	Alpha/Beta	Multi-peptide-supported	94	6	2	24	7.4	8.31	Deamidated [R2]; Oxidation [C12]	
B8XH01_v19	Lipolysis-activating peptide 1-alpha chain OS=*Buthus israelis* OX=2899555 PE=2 SV=1	*Buthus israelis*	Enzymes & accessory	Multi-peptide-supported	93	9	3	286	8.8	7.93	Methyl [S1; D2; N6; N10; T11; K15; T19; I20; E23; D25; Q28; K29; I30; K32; H34; Q37; D55; S57; I58; D60; K63]; Phospho [Y7]	
B8XH46	Potassium channel toxin alpha-KTx 9.8 OS=*Buthus israelis* OX=2899555 PE=3 SV=1	*Buthus israelis*	Potassium channel (KScTx)	Multi-peptide-supported	93	2	1	23	3	5.77		
P84744	Potassium channel toxin alpha-KTx 9.5 OS=*Buthus occitanus tunetanus* OX=6871 PE=1 SV=1	*Buthus occitanus tunetanus*	Potassium channel (KScTx)	Multi-peptide-supported	93	2	1	25	2.9	5.62		
B8XH01_v26	Lipolysis-activating peptide 1-alpha chain OS=*Buthus israelis* OX=2899555 PE=2 SV=1	*Buthus israelis*	Enzymes & accessory	Multi-peptide-supported	86	8	2	188	8.8	7.61	Deamidated [R64]; Methyl [S1; D2; N6; N10; T11; K15; T19; I20; E23; D25; Q28; K29; I30; K32; H34; Q37; H65]; Phospho [Y7]	
P0DJH8_v_WLGR	Alpha-toxin Bu1 OS=*Buthacus macrocentrus* OX=1143368 PE=1 SV=1	*Buthacus macrocentrus*	Insect/Mammal-specific	Multi-peptide-supported	84	6	2	28	7.5	8.48	Phospho [Y43]; Methyl [N45; C47; C49; L52]	
P01488_V	Alpha-toxin Bot1_V OS=*Buthus occitanus tunetanus_*V OX=6871 PE=1 SV=2	*Buthus occitanus tunetanus*	Insect/Mammal-specific	Multi-peptide-supported	80	5	4	10	7.3	7.55	Methyl [C48; K49; D50]; Oxidation [C48]; Phospho [Y42]	
Q17254_v	Alpha-insect toxin Bot14_V OS=*Buthus occitanus tunetanus*_v OX=6871 PE=2 SV=1	*Buthus occitanus tunetanus*	Insect/Mammal-specific	Multi-peptide-supported	70	5	5	16	7.6	8.15		

Accession	Description	BLAST best-hit organism	Functional category	Confidence tier	Coverage [%]	# Peptides	# Unique Peptides	# PSMs	MW [kDa]	calc. pI	Modifications	Comment/validation note
P17728	Alpha-insect toxin LqhaIT OS=*Leiurus hebraeus* OX=2899558 PE=1 SV=2	*Leiurus hebraeus*	Insect/Mammal-specific	Multi-peptide-supported	67	4	2	21	7.5	8.31	Methyl [I57; R58]	
B8XH07	Putative depressant toxin Tx131 (Fragment) OS=*Buthus israelis* OX=2899555 PE=2 SV=1	*Buthus israelis*	Putative	Tentative	66	1	1	2	4.1	5.02		
Q9UAC9_v2	Beta-toxin BmKAS_v2 OS=*Olivierus martensii*_v2 OX=34649 PE=1 SV=1	*Olivierus martensii*	Insect/Mammal-specific	Multi-peptide-supported	65	4	1	11	7.7	8.1	Methyl [L42; C46; Q47]	
P01486_v2	Alpha-toxin Bot11 OS=*Buthus occitanus tunetanus*_v2 OX=6871 PE=1 SV=1	*Buthus occitanus tunetanus*	Insect/Mammal-specific	Multi-peptide-supported	62	5	4	20	7.5	8.12	Methyl [N19]; Oxidation [C26]	
P84646_V	Alpha-toxin OD1_V OS=*Odontobuthus doriae*_V OX=342590 PE=1 SV=1	*Odontobuthus doriae*	Insect/Mammal-specific	Multi-peptide-supported	58	5	1	25	7.3	7.87		
C0HJQ2	Potassium channel toxin alpha-KTx 19.2 OS=*Buthus occitanus tunetanus* OX=6871 PE=1 SV=1	*Buthus occitanus tunetanus*	Potassium channel (KScTx)	Multi-peptide-supported	55	2	2	4	3.4	8.73	Oxidation [C3]	
B8XH44	Potassium channel toxin alpha-KTx 27.1 OS=*Buthus israelis* OX=2899555 PE=3 SV=1	*Buthus israelis*	Potassium channel (KScTx)	Multi-peptide-supported	54	2	2	3	5.6	8.66	Phospho [S7]; Methyl [D3; C18]	
ACJ23112.1	putative beta-like toxin Tx814 [*Buthus israelis*]	*Buthus israelis*	Putative	Multi-peptide-supported	52	7	6	201	11.1	8.18	Oxidation [C88]; Methyl [E45; E47; C49; E74; D75; K76; S79; T89]	
ACJ23099.1	putative alpha toxin Tx613 [*Buthus israelis*]	*Buthus israelis*	Putative	Multi-peptide-supported	52	5	1	23	9.5	7.53		
P86406_V1	MeuNaTxbeta-1 OS=*Mesobuthus eupeus* OX=34648 PE=1 SV=2	*Mesobuthus eupeus*	Insect/Mammal-specific	Multi-peptide-supported	50	3	1	5	7.8	7.53		
A0A1L2FZD4	Potassium channel toxin alpha-KTx 8.8 OS=*Orthochirus scrobiculosus* OX=6892 PE=1 SV=1	*Orthochirus scrobiculosus*	Potassium channel (KScTx)	Tentative	48	1	1	5	3.2	5.1		
ACJ23088.1	putative alpha toxin Tx2 [*Buthus israelis*]	*Buthus israelis*	Putative	Multi-peptide-supported	48	3	2	6	9.6	7.5		

Accession	Description	BLAST best-hit organism	Functional category	Confidence tier	Coverage [%]	# Peptides	# Unique Peptides	# PSMs	MW [kDa]	calc. pI	Modifications	Comment/validation note
P59896	Toxin BomPI OS=*Buthus occitanus mardochei* OX=6869 PE=1 SV=1	*Buthus mardochei*	Alpha/Beta	Multi-peptide-supported	47	2	1	4	7.4	7.55		
P0C292	Toxin Acra1 OS=*Androctonus crassicauda* OX=122909 PE=1 SV=1	*Androctonus crassicauda*	Alpha/Beta	Tentative	45	1	1	1	4.1	7.06		
P59863	Beta-toxin BotIT2 OS=*Buthus occitanus tunetanus* OX=6871 PE=1 SV=1	*Buthus occitanus tunetanus*	Insect/Mammal-specific	Multi-peptide-supported	43	3	3	7	6.9	4.84		
P86399	Neurotoxin lambda-MeuTx OS=*Mesobuthus eupeus* OX=34648 PE=1 SV=2	*Mesobuthus eupeus*	Alpha/Beta	Tentative	43	1	1	1	4.2	8		
D5HR51	Alpha-toxin Ac2 OS=*Androctonus crassicauda* OX=122909 PE=3 SV=1	*Androctonus crassicauda*	Insect/Mammal-specific	Multi-peptide-supported	42	2	1	11	7.4	8.47		
B8XH24	Putative chloride channel toxin Tx32 OS=*Buthus israelis* OX=2899555 PE=2 SV=1	*Buthus israelis*	Putative	Tentative	38	1	1	1	4.3	8.32		
A0A0U4QF63_v2	Sodium channel toxin NaTx7_v2 OS=*Odontobuthus doriae* OX=342590 GN=NaTx7 PE=2 SV=1	*Odontobuthus doriae*	Sodium channel (NaScTx)	Multi-peptide-supported	38	5	4	229	8.7	8.54	Oxidation [C66]; Methyl [D53; T67]	
B8XH19	Putative depressant toxin Tx695 OS=*Buthus israelis* OX=2899555 PE=2 SV=1	*Buthus israelis*	Putative	Tentative	37	1	1	1	6.9	8.65		
Q56TT9	Alpha-insect toxin BjaIT OS=*Hottentotta judaicus* OX=6863 PE=1 SV=1	*Hottentotta judaicus*	Insect/Mammal-specific	Multi-peptide-supported	35	2	2	5	7	7.87		
P0CH57	Potassium channel toxin MeuTXKbeta3-meucin-24 OS=*Mesobuthus eupeus* OX=34648 PE=1 SV=1	*Mesobuthus eupeus*	Potassium channel (KScTx)	Multi-peptide-supported	35	2	1	15	7.6	8.48		
B8XGZ2	Putative alpha toxin Tx644 OS=*Buthus israelis* OX=2899555 PE=2 SV=1	*Buthus israelis*	Putative	Multi-peptide-supported	34	2	2	4	7	8.31		
B8XH47	Potassium channel toxin alpha-KTx Tx790 OS=*Buthus israelis* OX=2899555 PE=2 SV=1	*Buthus israelis*	Potassium channel (KScTx)	Tentative	34	1	1	2	5.1	5.19		
Q8I0L5	Potassium channel toxin alpha-KTx 15.2 OS=*Olivierus martensii* OX=34649 PE=1 SV=1	*Olivierus martensii*	Potassium channel (KScTx)	Tentative	32	1	1	2	3.8	8.53		

Accession	Description	BLAST best-hit organism	Functional category	Confidence tier	Coverage [%]	# Peptides	# Unique Peptides	# PSMs	MW [kDa]	calc. pI	Modifications	Comment/validation note
B8XH40	Potassium channel toxin BuTXK-beta OS=*Buthus israelis* OX=2899555 PE=3 SV=1	*Buthus israelis*	Potassium channel (KScTx)	Multi-peptide-supported	30	2	2	5	7.1	8.27		
B8XH30	Potassium channel toxin alpha-KTx Tx308_v OS=*Buthus israelis*_v OX=2899555 PE=2 SV=1	*Buthus israelis*	Potassium channel (KScTx)	Tentative	30	1	1	2	5.3	8.28		
P04098	Neurotoxin 8 (Fragment) OS=*Buthus occitanus tunetanus* OX=6871 PE=1 SV=1	*Buthus occitanus tunetanus*	Alpha/Beta	Tentative	28	1	1	4	4.1	6.24		
Q9NJC4	Toxin BmKaTx17 (Fragment) OS=*Olivierus martensii* OX=34649 PE=2 SV=1	*Olivierus martensii*	Alpha/Beta	Tentative	27	1	1	2	7.2	7.09		
C0HM60	Toxin Bot33 OS=*Buthus occitanus tunetanus* OX=6871 PE=1 SV=2	*Buthus occitanus tunetanus*	Alpha/Beta	Tentative	26	1	1	7	4	8.72		
AMX81449.1	potassium channel toxin meuK28-2 [*Mesobuthus eupeus*]	*Mesobuthus eupeus*	Potassium channel (KScTx)	Multi-peptide-supported	26	2	1	14	10.4	8.24		
B8XH45	Potassium channel toxin alpha-KTx Tx773 OS=*Buthus israelis* OX=2899555 PE=2 SV=1	*Buthus israelis*	Potassium channel (KScTx)	Tentative	25	1	1	1	5.1	8.27		
A0A7T9L322	Hyaluronidase OS=*Androctonus crassicauda* OX=122909 GN=Hyal PE=2 SV=1	*Androctonus crassicauda*	Enzymes & accessory	Multi-peptide-supported	25	10	10	82	47.5	8.37	Methyl [S38; I39; D300; T375; S376; K378]	
P55903	Beta-insect depressant toxin BotIT4 OS=*Buthus occitanus tunetanus* OX=6871 PE=1 SV=1	*Buthus occitanus tunetanus*	Insect/Mammal-specific	Multi-peptide-supported	25	2	2	5	6.8	5.31		
Q9BLM4	Toxin AahP1005 OS=*Androctonus australis* OX=6858 PE=2 SV=1	*Androctonus australis*	Alpha/Beta	Multi-peptide-supported	24	2	2	13	7.4	8.62		
P68721	Beta-insect excitatory toxin LqhIT1a OS=*Leiurus hebraeus* OX=2899558 PE=3 SV=1	*Leiurus hebraeus*	Insect/Mammal-specific	Tentative	23	1	1	1	7.9	7.87		
P0CI98	Neurotoxin 213 OS=*Lychas mucronatus* OX=172552 PE=3 SV=1	*Lychas mucronatus*	Alpha/Beta	Tentative	22	1	1	1	8.5	7.93	Phospho [S9; S13]	
AMX81463.1	sodium channel toxin meuNaTx-5 [*Mesobuthus eupeus*]	*Mesobuthus eupeus*	Sodium channel (NaScTx)	Multi-peptide-supported	22	3	2	14	10.1	8.91	Methyl [N76]	

Accession	Description	BLAST best-hit organism	Functional category	Confidence tier	Coverage [%]	# Peptides	# Unique Peptides	# PSMs	MW [kDa]	calc. pI	Modifications	Comment/validation note
AAA69557.1	pre-Bmk1 neurotoxin [*Olivierus martensii*]	*Olivierus martensii*	Alpha/Beta	Tentative	22	1	1	3	8.9	7.88		
B8XH29	Putative potassium channel toxin Tx260 OS=*Buthus israelis* OX=2899555 PE=2 SV=1	*Buthus israelis*	Putative	Tentative	21	1	1	1	4.2	9.03	Methyl [D16]	
A0A2I9LP14	Hemocyanin subunit F OS=*Centruroides hentzi* OX=88313 PE=3 SV=1	*Centruroides hentzi*	Enzymes & accessory	Multi-peptide-supported	20	14	9	46	71.9	5.96	Methyl [I10; I11; I151; L152; E155; K340; N342; I357; D598; R599]	
B8XH20	Putative depressant toxin Tx700 OS=*Buthus israelis* OX=2899555 PE=2 SV=1	*Buthus israelis*	Putative	Tentative	19	1	1	2	6.7	7.87		
P80476	Hemocyanin AA6 chain OS=*Androctonus australis* OX=6858 PE=1 SV=1	*Androctonus australis*	Enzymes & accessory	Multi-peptide-supported	19	13	3	81	71.7	5.82	Methyl [Q111; E112; D116; D599; N614; D619; I620; K621]	
ACJ23118.1	putative depressant toxin Tx273 [*Buthus israelis*]	*Buthus israelis*	Putative	Multi-peptide-supported	17	2	2	10	9.7	8.76		
B8XH12	Putative depressant toxin Tx438 OS=*Buthus israelis* OX=2899555 PE=2 SV=1	*Buthus israelis*	Putative	Tentative	17	1	1	3	6.6	8.1		
P59290	Potassium channel toxin alpha-KTx 3.9 OS=*Buthus occitanus tunetanus* OX=6871 GN=KTX3 PE=1 SV=1	*Buthus occitanus tunetanus*	Potassium channel (KScTx)	Tentative	16	1	1	1	4	9		
A0A2I9LP22	Hemocyanin AA6 chain OS=*Centruroides hentzi* OX=88313 PE=3 SV=1	*Centruroides hentzi*	Enzymes & accessory	Multi-peptide-supported	16	11	2	73	72	5.77	Methyl [Q112; E113; D117; D294; D296; D600]	
A0AA50AAI4	Phospholipase A2 OS=*Androctonus crassicauda* OX=122909 PE=2 SV=1	*Androctonus crassicauda*	Enzymes & accessory	Multi-peptide-supported	16	6	6	31	25	6.95		
E7CZZ1	Sodium channel neurotoxin MeuNaTxalpha-12 (Fragment) OS=*Mesobuthus eupeus* OX=34648 PE=2 SV=1	*Mesobuthus eupeus*	Sodium channel (NaScTx)	Tentative	14	1	1	4	7.2	8.12		
C6H100	Hemocyanin subunit 5a OS=*Pandinus imperator* OX=55084 GN=hc5a PE=2 SV=1	*Pandinus imperator*	Enzymes & accessory	Multi-peptide-supported	13	11	6	71	71.9	6.44	Methyl [T124; H333; I357; D464; E465; L466; N468; R469; D598]; Deamidated [R469]	

Accession	Description	BLAST best-hit organism	Functional category	Confidence tier	Coverage [%]	# Peptides	# Unique Peptides	# PSMs	MW [kDa]	calc. pI	Modifications	Comment/validation note
XHB14016.1	sodium channel scorpion toxin pMeNaTx8-6 [*Mesobuthus eupeus*]	*Mesobuthus eupeus*	Sodium channel (NaScTx)	Tentative	11	1	1	17	11.2	8.54	Methyl [H84; H85; T89]	
B8XH25	Putative chloride channel toxin Tx357 (Fragment) OS=*Buthus israelis* OX=2899555 PE=2 SV=1	*Buthus israelis*	Putative	Tentative	10	1	1	3	7.7	8.68		
A0A1E1WVL6	Putative hemocyanin subunit OS=*Tityus obscurus* OX=1221240 PE=4 SV=1	*Tityus obscurus*	Enzymes & accessory	Multi-peptide-supported	10	7	5	54	73.2	6.71	Methyl [S504; N505; L506; I507; D609]	
AIX87687.1	potassium channel blocker AbKTx-10 [*Androctonus bicolor*]	*Androctonus bicolor*	Potassium channel (KScTx)	Tentative	10	1	1	2	10.1	8.75	Methyl [T41]	
B8XH32	Putative potassium channel toxin Tx479 OS=*Buthus israelis* OX=2899555 PE=2 SV=1	*Buthus israelis*	Putative	Tentative	10	1	1	1	6.8	8.41		
O77091	Beta-insect excitatory toxin BmK IT-AP OS=*Olivierus martensii* OX=34649 PE=1 SV=1	*Olivierus martensii*	Insect/Mammal-specific	Tentative	8	1	1	17	8.2	5.01		

Column definitions — # Peptides: distinct peptide sequences matched to the protein; # Unique Peptides: peptides assigned uniquely to a single protein group (proteotypic); # PSMs: peptide-spectrum matches; Modifications: PTM sites localised at a Proteome Discoverer site probability ≥ 0.75; Confidence tier: Multi-peptide-supported (≥ 2 peptides at FDR < 1%) or Tentative (single-peptide identification); BLAST best-hit organism: source organism of the closest database homolog; Functional category: toxin family assignment (Tables S8–S13).

Table S6. Sequence coverage of major protein variants compared with their wild-type counterparts, as identified by Proteome Discoverer.

Accession number	Description	Coverage WT (%)	Coverage variant (%)
**P60257_V+V2_F>A17**	Toxin Boma6c OS=*Buthus occitanus mardochei* OX=6869 PE=3 SV=1	76	94
**B8XH01_v19**	Lipolysis-activating peptide 1-alpha chain OS=*Buthus israelis* OX=2899555 PE=2 SV=1	44	93
**P0DJH8_v_WLGR**	Alpha-toxin Bu1 OS=*Buthacus macrocentrus* OX=1143368 PE=1 SV=1	39	84
**P01488_V**	Alpha-toxin Bot1_V OS=*Buthus occitanus tunetanus*_V OX=6871 PE=1 SV=2	20	80
**Q17254_v**	Alpha-insect toxin Bot14_V OS=*Buthus occitanus tunetanus*_v OX=6871 PE=2 SV=1	59	70
**Q9UAC9_v2**	Beta-toxin BmKAS_v2 OS=*Olivierus martensii*_v2 OX=34649 PE=1 SV=1	24	65
**P01486_v2**	Alpha-toxin Bot11 OS=*Buthus occitanus tunetanus*_v2 OX=6871 PE=1 SV=1	46	62
**P84646_V**	Alpha-toxin OD1_V OS=*Odontobuthus doriae*_V OX=342590 PE=1 SV=1	46	58
**P86406_V1**	MeuNaTxbeta-1 OS=*Mesobuthus eupeus* OX=34648 PE=1 SV=2	12	50
**A0A0U4QF63_v2**	Sodium channel toxin NaTx7_v2 OS=*Odontobuthus doriae* OX=342590 GN=NaTx7 PE=2 SV=1	7	38

Column definitions — WT, wild-type; Coverage WT (%) and Coverage variant (%): sequence coverage of the wild-type and constructed-variant forms, respectively.

Table S7. Detailed comparison of wild-type and variant sequence coverage, including Casanovo *de novo* sequencing results and screenshots of the alignment regions identified by Proteome Discoverer.

Wildtype and Variant sequence rows reproduce the original alignment screenshots (sequence regions highlighted by Proteome Discoverer); Casanovo rows give the de novo sequencing read-out for the corresponding region, where available.

**Accession number: P60257_V+V2_F>A17 Description: Toxin Boma6c OS=*Buthus occitanus mardochei* OX=6869 PE=3 SV=1**	
**Wildtype**	VRDAYIAQNY NCVYTCFKDA HCNDLCTKNG ASSGYCQWAG KYGNACWCYA LPDNVPIRIP GKCHRK
**Casanovo**	YGNACWCYA LPDNVP L R L P GK NCVYTC A K DA Y CNDLCTK
**Variant sequence**	VRDAYIAQNY NCVYTCAKDA YCNDLCTKNG ASSGYCQWAG KYGNACWCYA LPDNVPLRLP GKCHRK
**Accession number: B8XH01_v19 Description: Lipolysis-activating peptide 1-alpha chain OS=*Buthus israelis* OX=2899555 PE=2 SV=1**	
**Wildtype**	MMKLVLFGII VILFSMIGSI HGSDPPGNYP LNTYGNKYAC TILGENDFCQ KICKVHGVQY GYCFNSRCWC EYLEEKDVNI WDAVKRHCKN TILYPKGK
**Sequence of conflict (Uniprot)**	Sequence conflict 79 in Ref. 1; ACJ23106/ACJ23108/ACJ23112 Curated<br>**Sequence:** N → S
**Variant sequence**	SDPPGNYPLN TYGNKYACTI LGENDFCQKI CKVHGVQYGY CFNSRCWCEY LEEKDVSIWD AVKRHCKNTI LYPKGK
**Accession number: P0DJH8_v_WLGR Description: Alpha-toxin Bu1 OS=*Buthacus macrocentrus* OX=1143368 PE=1 SV=1**	
**Wildtype**	GVRDAYIADD KNCVYTCAKN SYCNTECTKN GAESGYCQWL GKYGNGCWCI KLPDKVPIRI PGRCRGR
**Casanovo**	NGAESGYCQWLG R
**Variant sequence**	GVRDAYIADD KNCVYTCAKN SYCNTECTKN GAESGYCQWL GRYGNGCWCI KLPDKVPIRI PGRCRGR
**Accession number: P01488_V Description: Alpha-toxin Bot1_V OS=*Buthus occitanus tunetanus* OX=6871 PE=1 SV=2**	
**Wildtype**	GRDAYIAQPE NCVYECAQNS YCNDLCTKNG ATSGYCQWLG KYGNACWCKD LPDNVPIRIP GKCHF
**Casanovo**	RENYLAQPENCVYECA K YGNACWCYALPDNVP L R L PGK
**Variant sequence**	GRDAYIAQPE NCVYECAKNS YCNDLCTKNG ATSGYCQWLG KYGNACWCKD LPDNVPLRLP GKCHF
**Accession number: Q17254_v Description: Alpha-insect toxin Bot14_V OS=*Buthus occitanus tunetanus* OX=6871 PE=2 SV=1**	
**Wildtype**	MSSLMISTAM KGKAPYRQVR DGYIAQPHNC AYHCLKISSG CDTLCKENGA TSGHCGHKSG HGSACWCKDL PDKVGIIVHG EKCHR
**Casanovo**	GATSGHCG LLAR
**Variant sequence**	VRDGYIAQPH NCAYHCLKIS SGCDTLCKEN GATSGHCGLL ARHKSGHGSA CWCKDLPDKV GIIVHGEKCH R
**Accession number : Q9UAC9_v2 Description : Beta-toxin BmKAS_v2 OS=*Olivierus martensii*_v2 OX=34649 PE=1 SV=1**	
**Wildtype**	MKTVIFLIVS SLLLIGVKTD NGYLLDKYTG CKVWCVINNE SCNSECKIRG GYYGYCYFWK LACFCQGARK SELWNYNTNK CNGKL
**Casanovo**	VWCV L NNE ADC SECK
**Variant sequence**	DNGYLLDKYT GCKVWCVLNN EADCSECKIR GGYYGYCYFW KLACFCQGAR KSELWNYNTN KCNGKL
**Accession number : P01486_v2 Description : Alpha-toxin Bot11 OS=*Buthus occitanus tunetanus*_v2 OX=6871 PE=1 SV=1**	
**Wildtype**	LKDGYIVDDR NCTYFCGTNA YCNEECVKLK GESGYCQWVG RYGNACWCYK LPDHVRTVQA GRCRS
**Casanovo**	NCTYFCG R QTGNEEC N K
**Variant sequence**	LKDGYIVDDR NCTYFCGRNA YCNEECNKLK GESGYCQWVG RYGNACWCYK LPDHVRTVQA GRCRS
**Accession number : P84646_V Description : Alpha-toxin OD1_V OS=*Odontobuthus doriae*_V OX=342590 PE=1 SV=1**	
**Wildtype**	GVRDAYIADD KNCVYTCASN GYCNTECTKN GAESGYCQWI GRYGNACWCI KLPDEVPIRI PGKCR
**Casanovo**	NCVYTCA K

**Variant sequence**	C C D C C C<br>GVRDAYLADD KNCVYTCAKN GYCNTECTKN GAESGYCQWI GRYGNACWCI KLPDEVPIRI PGKCR
**Accession number : P86406_V1 Description : MeuNaTxbeta-1 OS=*Mesobuthus eupeus* OX=34648 PE=1 SV=2**	
**Wildtype**	C C<br>MMKIIIFLIV SSLVLIGVKT DNGYLLDKYT GCKVWCVINN ESCNSECKIR RGNYGYCYFW KLACYCEGAP KSELWHYETN KCNGRM
**Casanovo**	VWCV L NNE ADC SECK
**Variant sequence**	C C C C C<br>DNGYLLDKYT GCKVWCVLNN EADCSECKIR RGNYGYCYFW KLACYCEGAP KSELWHYETN KCNGRM
**Accession number : A0A0U4QF63_v2 Description : Sodium channel toxin NaTx7_v2 OS=*Odontobuthus doriae* OX=342590 GN=NaTx7 PE=2 SV=1**	
**Wildtype**	C C<br>MKLLLFGIIL TLFSLIGSIG GADVPGNYPA NVYGKKYLCG DLGENAYCRK VCKLHGVSYG YCFQFKCWCE YLKDKDISIW NAIKNHCTNT FLYPNGK
**Casanovo**	NHCTNT L LYP K
**Variant sequence**	OO<br>COC M OD D CMDO<br>ADVPGNYPAN VYGKKYLCGD LGENAYCRKV CKLHGVSYGY CFQFKCWCEY LKDKDISIWN AIKNHCTNTL LYPKGK

Validation of de novo–derived variants. The Casanovo de novo sequences listed here were used to construct candidate sequence variants, which were appended to the reference database and re-identified with Proteome Discoverer 3.2 (Sequest HT search, Percolator validation). A variant was retained only when its region was confirmed by Proteome Discoverer under uniform criteria: precursor mass tolerance 10 ppm, fragment mass tolerance 0.05 Da, Percolator q-value validation at a strict target FDR of 0.01 (< 1%), and a maximum ΔCn of 0.05. For each variant, the accompanying screenshot shows the corresponding Proteome Discoverer–identified spectrum confirming the assignment. Complete Proteome Discoverer and Casanovo parameters are provided in Supplementary Data S1.

Table S8. Categorization of channel toxins identified in the BotG50 fraction — Potassium channel toxins.

Each identified component is annotated with a functional evidence level. Predicted: assignment based on sequence homology, conserved cysteine framework or database annotation only. Inferred: function supported by a closely related, experimentally characterised homologue (literature consensus), the exact BotG50 sequence not itself tested. Validated: direct functional evidence available for the same B. occitanus tunetanus toxin or an identical mature sequence. No component is described as functionally validated on the basis of the present proteomic analysis alone.

Accession	Description	Evidence level	Evidence basis	Supporting reference
B8XH46	Potassium channel toxin alpha-KTx 9.8 OS=*Buthus israelis* OX=2899555 PE=3 SV=1	**Predicted**	Homology to alpha-KTx family + conserved cysteine framework; transcriptome-derived	—
P84744	Potassium channel toxin alpha-KTx 9.5 OS=*Buthus occitanus tunetanus* OX=6871 PE=1 SV=1	**Validated**	Same *B. o. tunetanus* toxin (Kbot1); block of Kv1.1/1.2/1.3 and SK channels	Mahjoubi-Boubaker et al., Peptides 2004, 25, 637-645 [main ref 31]
C0HJQ2	Potassium channel toxin alpha-KTx 19.2 OS=*Buthus occitanus tunetanus* OX=6871 PE=1 SV=1	**Predicted**	Homology to alpha-KTx family; no functional assay for this exact toxin	—
B8XH44	Potassium channel toxin alpha-KTx 27.1 OS=*Buthus israelis* OX=2899555 PE=3 SV=1	**Predicted**	Homology to alpha-KTx family; transcriptome-derived	—
A0A1L2FZD4	Potassium channel toxin alpha-KTx 8.8 OS=*Orthochirus scrobiculosus* OX=6892 PE=1 SV=1	**Predicted**	Homology to alpha-KTx family	—
B8XH47	Potassium channel toxin alpha-KTx Tx790 OS=*Buthus israelis* OX=2899555 PE=2 SV=1	**Predicted**	Homology to alpha-KTx family; transcriptome-derived	—
Q8I0L5	Potassium channel toxin alpha-KTx 15.2 OS=*Olivierus martensii* OX=34649 PE=1 SV=1	**Predicted**	Homology to alpha-KTx family	—
B8XH40	Potassium channel toxin BuTXK-beta OS=*Buthus israelis* OX=2899555 PE=3 SV=1	**Predicted**	Homology to beta-KTx family; transcriptome-derived	—
B8XH30	Potassium channel toxin alpha-KTx Tx308_v OS=*Buthus israelis*_v OX=2899555 PE=2 SV=1	**Predicted**	Homology to alpha-KTx family; constructed variant	—
P0CH57	Potassium channel toxin MeuTXKbeta3-meucin-24 OS=*Mesobuthus eupeus* OX=34648 PE=1 SV=1	**Predicted**	Homology to beta-KTx family	—
AMX81449.1	potassium channel toxin meuK28-2 [*Mesobuthus eupeus*]	**Predicted**	Homology to K-channel toxin family	—
B8XH45	Potassium channel toxin alpha-KTx Tx773 OS=*Buthus israelis* OX=2899555 PE=2 SV=1	**Predicted**	Homology to alpha-KTx family; transcriptome-derived	—
P59290	Potassium channel toxin alpha-KTx 3.9 OS=*Buthus occitanus tunetanus* OX=6871 GN=KTX3 PE=1 SV=1	**Validated**	Same *B. o. tunetanus* toxin (KTX3); K+-channel ligand, in vivo toxicity + synaptosomal binding	Meki et al., Toxicon 2000, 38, 105-111 [main ref 32]
AIX87687.1	potassium channel blocker AbKTx-10 [*Androctonus bicolor*]	**Predicted**	Homology to K-channel blocker family	—

Table S9. Categorization of channel toxins identified in the BotG50 fraction — Sodium channel toxins.

Accession	Description	Evidence level	Evidence basis	Supporting reference
A0A0U4QF63_v2	Sodium channel toxin NaTx7_v2 OS=*Odontobuthus doriae* OX=342590 GN=NaTx7 PE=2 SV=1	**Predicted**	Homology to NaScTx family; constructed variant	—
AMX81463.1	sodium channel toxin meuNaTx-5 [*Mesobuthus eupeus*]	**Predicted**	Homology to NaScTx family	—
E7CZZ1	Sodium channel neurotoxin MeuNaTxalpha-12 (Fragment) OS=*Mesobuthus eupeus* OX=34648 PE=2 SV=1	**Predicted**	Homology to NaScTx family (fragment)	—
XHB14016.1	sodium channel scorpion toxin pMeNaTx8-6 [*Mesobuthus eupeus*]	**Predicted**	Homology to NaScTx family	—

Table S10. Categorization of channel toxins identified in the BotG50 fraction — Alpha/Beta toxins.

Accession	Description	Evidence level	Evidence basis	Supporting reference
P60257_V+V2_F>A17	Toxin Boma6c_V OS=*Buthus occitanus mardochei*_V OX=6869 PE=3 SV=1	**Predicted**	Homology to alpha/beta-toxin family; Casanovo-constructed variant	—
P59896	Toxin BomPI OS=*Buthus occitanus mardochei* OX=6869 PE=1 SV=1	**Predicted**	Homology to alpha/beta-toxin family	—
P0C292	Toxin Acra1 OS=*Androctonus crassicauda* OX=122909 PE=1 SV=1	**Predicted**	Sequence only (cDNA + MS); function By similarity	—
P86399	Neurotoxin lambda-MeuTx OS=*Mesobuthus eupeus* OX=34648 PE=1 SV=2	**Predicted**	Homology; putative Kv function only in an unpublished UniProt submission (no primary paper)	—
P04098	Neurotoxin 8 (Fragment) OS=*Buthus occitanus tunetanus* OX=6871 PE=1 SV=1	**Inferred**	Isolated *B. o. tunetanus* toxin, mammal-lethal; Nav site-3 mechanism from literature consensus (no direct EP)	Martin & Rochat, Toxicon 1984, 22, 279-291 [main ref 42]
Q9NJC4	Toxin BmKaTx17 (Fragment) OS=*Olivierus martensii* OX=34649 PE=2 SV=1	**Predicted**	Homology to alpha/beta-toxin family	—
C0HM60	Toxin Bot33 OS=*Buthus occitanus tunetanus* OX=6871 PE=1 SV=2	**Validated**	Same *B. o. tunetanus* toxin; experimentally tested - no Kv activity up to 1 uM, non-toxic to mice	ElFessi et al., Molecules 2022, 27, 7278 [main ref 28]
Q9BLM4	Toxin AahP1005 OS=*Androctonus australis* OX=6858 PE=2 SV=1	**Predicted**	Sequence only (cDNA); function By similarity	—
P0CI98	Neurotoxin 213 OS=*Lychas mucronatus* OX=172552 PE=3 SV=1	**Predicted**	Homology to alpha/beta-toxin family	—
AAA69557.1	pre-Bmk1 neurotoxin [*Olivierus martensii*]	**Predicted**	Homology (precursor)	—

Table S11. Categorization of channel toxins identified in the BotG50 fraction — Putative toxins.

Accession	Description	Evidence level	Evidence basis	Supporting reference
B8XH07	Putative depressant toxin Tx131 (Fragment) OS=*Buthus israelis* OX=2899555 PE=2 SV=1	**Predicted**	Putative assignment by database annotation / homology only	—
B8XH24	Putative chloride channel toxin Tx32 OS=*Buthus israelis* OX=2899555 PE=2 SV=1	**Predicted**	Putative assignment by database annotation / homology only	—
B8XH20	Putative depressant toxin Tx700 OS=*Buthus israelis* OX=2899555 PE=2 SV=1	**Predicted**	Putative assignment by database annotation / homology only	—
B8XH19	Putative depressant toxin Tx695 OS=*Buthus israelis* OX=2899555 PE=2 SV=1	**Predicted**	Putative assignment by database annotation / homology only	—
B8XH25	Putative chloride channel toxin Tx357 (Fragment) OS=*Buthus israelis* OX=2899555 PE=2 SV=1	**Predicted**	Putative assignment by database annotation / homology only	—
ACJ23118.1	putative depressant toxin Tx273 [*Buthus israelis*]	**Predicted**	Putative assignment by database annotation / homology only	—
B8XH12	Putative depressant toxin Tx438 OS=*Buthus israelis* OX=2899555 PE=2 SV=1	**Predicted**	Putative assignment by database annotation / homology only	—
B8XH29	Putative potassium channel toxin Tx260 OS=*Buthus israelis* OX=2899555 PE=2 SV=1	**Predicted**	Putative assignment by database annotation / homology only	—
B8XH32	Putative potassium channel toxin Tx479 OS=*Buthus israelis* OX=2899555 PE=2 SV=1	**Predicted**	Putative assignment by database annotation / homology only	—
ACJ23099.1	putative alpha toxin Tx613 [*Buthus israelis*]	**Predicted**	Putative assignment by database annotation / homology only	—
ACJ23088.1	putative alpha toxin Tx2 [*Buthus israelis*]	**Predicted**	Putative assignment by database annotation / homology only	—
B8XGZ2	Putative alpha toxin Tx644 OS=*Buthus israelis* OX=2899555 PE=2 SV=1	**Predicted**	Putative assignment by database annotation / homology only	—
ACJ23112.1	putative beta-like toxin Tx814 [*Buthus israelis*]	**Predicted**	Putative assignment by database annotation / homology only	—

Table S12. Categorization of channel toxins identified in the BotG50 fraction — Insect/Mammal-specific neurotoxins.

Accession	Description	Evidence level	Evidence basis	Supporting reference
P0DJH8_v_WLGR	Alpha-toxin Bu1 OS=*Buthacus macrocentrus* OX=1143368 PE=1 SV=1	**Inferred**	Variant of characterised Bu1 (Nav alpha-toxin, DRG electrophysiology); cross-species	Caliskan et al., Toxicon 2012, 59, 408-415 [main ref 41]
P01488_V	Alpha-toxin Bot1_V OS=*Buthus occitanus tunetanus* OX=6871 PE=1 SV=2	**Inferred**	*B. o. tunetanus* alpha-toxin, mammal-lethal; Nav site-3 from literature consensus; constructed variant	Martin & Rochat, Toxicon 1984, 22, 279-291 [main ref 42]
Q17254_v	Alpha-insect toxin Bot14_V OS=*Buthus occitanus tunetanus*_v OX=6871 PE=2 SV=1	**Inferred**	Variant of characterised insect-specific Bot14 (recombinant insect toxicity)	Bouhaouala-Zahar et al., Eur. J. Biochem. 1996, 238, 653-660 [main ref 34]
P17728	Alpha-insect toxin LqhaIT OS=*Leiurus hebraeus* OX=2899558 PE=1 SV=2	**Inferred**	Characterised insect alpha-toxin (*Leiurus*); cross-species homologue, not identical sequence	Eitan et al., Biochemistry 1990, 29, 5941-5947 [main ref 35]
P01486_v2	Alpha-toxin Bot11 OS=*Buthus occitanus tunetanus*_v2 OX=6871 PE=1 SV=1	**Inferred**	*B. o. tunetanus* alpha-toxin, mammal-lethal; Nav from literature consensus; variant	Martin & Rochat, Toxicon 1984, 22, 279-291 [main ref 42]
P84646_V	Alpha-toxin OD1_V OS=*Odontobuthus doriae* OX=342590 PE=1 SV=1	**Inferred**	Variant of characterised OD1 (Nav1.7 activator); cross-species (*Odontobuthus*)	Jalali et al., FEBS Lett. 2005, 579, 4181-4186 [main ref 36]
D5HR51	Alpha-toxin Ac2 OS=*Androctonus crassicauda* OX=122909 PE=3 SV=1	**Predicted**	Sequence only (genomic DNA); function By similarity	—
Q56TT9	Alpha-insect toxin BjaIT OS=*Hottentotta judaicus* OX=6863 PE=1 SV=1	**Inferred**	Characterised insect-selective alpha-toxin (Buthotus); cross-species homologue	Arnon et al., Insect Biochem. Mol. Biol. 2005, 35, 187-195 [main ref 39]
Q9UAC9_v2	Beta-toxin BmKAS_v2 OS=*Olivierus martensii*_v2 OX=34649 PE=1 SV=1	**Inferred**	Variant of characterised BmK AS (Nav modulator); cross-species (*Olivierus*)	Li et al., J. Neurosci. Res. 2000, 61, 541-548 [main ref 40]
P86406_V1	MeuNaTxbeta-1 OS=*Mesobuthus eupeus* OX=34648 PE=1 SV=2	**Predicted**	Homology to NaScTx beta family; variant	—
P59863	Beta-toxin BotIT2 OS=*Buthus occitanus tunetanus* OX=6871 PE=1 SV=1	**Validated**	Same *B. o. tunetanus* toxin; insect Na-channel activation, cockroach axon electrophysiology	Borchani et al., Eur. J. Biochem. 1996, 241, 525-532 [main ref 33]
P55903	Beta-insect depressant toxin BotIT4 OS=*Buthus occitanus tunetanus* OX=6871 PE=1 SV=1	**Validated**	Same *B. o. tunetanus* toxin; depressant insect toxin, purification + toxicity + Na-channel binding (depressant-class electrophysiology)	Borchani et al., Toxicon 1997, 35, 365-382 [main ref 23]
P68721	Beta-insect excitatory toxin LqhIT1a OS=*Leiurus hebraeus* OX=2899558 PE=3 SV=1	**Inferred**	Prototype excitatory toxin Lqh-xtrIT characterised (homologue); this entry By similarity	Froy et al., J. Biol. Chem. 1999, 274, 5769-5776 [main ref 37]
O77091	Beta-insect excitatory toxin BmK IT-AP OS=*Olivierus martensii* OX=34649 PE=1 SV=1	**Inferred**	Characterised excitatory insect / analgesic toxin (*Olivierus*); cross-species homologue	Xiong et al., Toxicon 1999, 37, 1165-1180 [main ref 38]

Table S13. Categorization of channel toxins identified in the BotG50 fraction — Enzymes and accessory venom proteins.

Accession	Description	Evidence level	Evidence basis	Supporting reference
B8XH01_v19	Lipolysis-activating peptide 1-alpha chain OS=*Buthus israelis* OX=2899555 PE=2 SV=1	**Predicted**	Homology to lipolysis-activating peptide; constructed variant	—
B8XH01_v26	Lipolysis-activating peptide 1-alpha chain OS=*Buthus israelis* OX=2899555 PE=2 SV=1	**Predicted**	Homology to lipolysis-activating peptide; constructed variant	—
A0A7T9L322	Hyaluronidase OS=*Androctonus crassicauda* OX=122909 GN=Hyal PE=2 SV=1	**Predicted**	Enzyme class by homology (glycosyl hydrolase); no activity assay	—
A0A2I9LP14	Hemocyanin subunit F OS=*Centruroides hentzi* OX=88313 PE=3 SV=1	**Predicted**	Homology to hemocyanin family	—
P80476	Hemocyanin AA6 chain OS=*Androctonus australis* OX=6858 PE=1 SV=1	**Predicted**	Homology to hemocyanin family	—
A0A2I9LP22	Hemocyanin AA6 chain OS=*Centruroides hentzi* OX=88313 PE=3 SV=1	**Predicted**	Homology to hemocyanin family	—
A0AA50AAI4	Phospholipase A2 OS=*Androctonus crassicauda* OX=122909 PE=2 SV=1	**Predicted**	Enzyme class by homology; no activity assay	—
C6H100	Hemocyanin subunit 5a OS=*Pandinus imperator* OX=55084 GN=hc5a PE=2 SV=1	**Predicted**	Homology to hemocyanin family	—
A0A1E1WVL6	Putative hemocyanin subunit OS=*Tityus obscurus* OX=1221240 PE=4 SV=1	**Predicted**	Homology to hemocyanin family	—

Supplementary references (functional evidence, Tables S8–S13) — included in the main reference list as [31]–[42]

Borchani et al. (Toxicon 1997; BotIT4) and ElFessi et al. (Molecules 2022; Bot33) correspond to main-text references [23] and [28].

31. Mahjoubi-Boubaker, B.; Crest, M.; Ben Khalifa, R.; El Ayeb, M.; Kharrat, R. Kbot1, a three disulfide bridges toxin from *Buthus occitanus tunetanus* venom highly active on both SK and Kv channels. Peptides 2004, 25, 637–645.

32. Meki, A.; Mansuelle, P.; Laraba-Djebari, F.; Oughideni, R.; Rochat, H.; Martin-Eauclaire, M.-F. KTX3, the kaliotoxin from *Buthus occitanus tunetanus* scorpion venom. Toxicon 2000, 38, 105–111.

33. Borchani, L.; Mansuelle, P.; Stankiewicz, M.; Grolleau, F.; Cestèle, S.; Karoui, H.; Lapied, B.; Rochat, H.; Pelhate, M.; El Ayeb, M. A new scorpion venom toxin paralytic to insects that affects Na+ channel activation. Eur. J. Biochem. 1996, 241, 525–532.

34. Bouhaouala-Zahar, B.; Ducancel, F.; Zenouaki, I.; Ben Khalifa, R.; Borchani, L.; Pelhate, M.; Boulain, J.-C.; El Ayeb, M.; Ménez, A.; Karoui, H. A recombinant insect-specific alpha-toxin of *Buthus occitanus tunetanus* scorpion. Eur. J. Biochem. 1996, 238, 653–660.

35. Eitan, M.; Fowler, E.; Herrmann, R.; Duval, A.; Pelhate, M.; Zlotkin, E. A scorpion venom neurotoxin paralytic to insects that affects sodium current inactivation. Biochemistry 1990, 29, 5941–5947.

36. Jalali, A.; Bosmans, F.; Amininasab, M.; et al. OD1, the first toxin isolated from the venom of Odonthobuthus doriae active on voltage-gated Na+ channels. FEBS Lett. 2005, 579, 4181–4186.

37. Froy, O.; Zilberberg, N.; Gordon, D.; et al. The putative bioactive surface of insect-selective scorpion excitatory neurotoxins. J. Biol. Chem. 1999, 274, 5769–5776.

38. Xiong, Y.-M.; Lan, Z.-D.; Wang, M.; et al. Molecular characterization of a new excitatory insect neurotoxin with an analgesic effect on mice from the scorpion *Buthus martensi* Karsch. Toxicon 1999, 37, 1165–1180.

39. Arnon, T.; Potikha, T.; Sher, D.; et al. BjαIT: a novel scorpion α-toxin selective for insects. Insect Biochem. Mol. Biol. 2005, 35, 187–195.

40. Li, Y.-J.; Liu, Y.; Ji, Y.-H. BmK AS: new scorpion neurotoxin binds to distinct receptor sites of mammal and insect voltage-gated sodium channels. J. Neurosci. Res. 2000, 61, 541–548.

41. Çalışkan, F.; Quintero-Hernández, V.; Restano-Cassulini, R.; et al. Turkish scorpion *Buthacus macrocentrus*: description of Bu1, a potent mammalian Na-channel alpha-toxin. Toxicon 2012, 59, 408–415.

42. Martin, M.-F.; Rochat, H. Purification of thirteen toxins active on mice from the venom of the North African scorpion *Buthus occitanus tunetanus*. Toxicon 1984, 22, 279–291.

Table S14. Excluded non-scorpion database cross-match.
A0A2Z6MCU4 — Knottin scorpion toxin-like domain-containing protein OS=*Trifolium subterraneum* OX=3900 GN=TSUD_18940 PE=4 SV=1. BLAST best-hit organism: *Trifolium subterraneum* (plant; non-scorpion). This entry was retrieved during the BLAST-guided homology-expansion stage, solely through the inhibitor cystine-knot (ICK / knottin) motif shared between scorpion toxins and plant knottins. It corresponds to a single-peptide, single-PSM identification (sequence coverage 17%; 1 peptide; 1 unique peptide; 1 PSM). It was excluded from the final BotG50 venom-component set and from the stage-wise result tables, as it does not represent a genuine venom component, and is reported here solely for transparency and reproducibility.

Supplementary Data S1 Proteome Discoverer parameter:
The FASTA database ScorpianVariants042326.fasta used in this Proteome Discoverer run corresponds to the final enriched reference database, compiled after all five expansion stages (initial UniProtKB retrieval, BLAST-guided homology expansion, and Casanovo-derived variant construction). The March 2026 access dates cited in the main text (Section 4.5.1–4.5.2) refer to the initial UniProtKB retrieval and BLAST expansion steps, respectively; the April 2026 file date reflects the completion of the iterative enrichment process.

Processing Step A: Workflow
Result name: BOT_G50_digest_nodilution_1µL
Description: Processing workflow for precursor-based quantification, using SequestHT with Multi Peptide Search and Percolator validation. Specify the FASTA database, labels used, and any additional modifications.
Workflow based on template: PWF_Hybrid_Precursor_Quan_and_LFQ_MPS_SequestScorpionPTM2_Percolator
Created with Discoverer version: 3.2.0.450

The workflow tree:

 |-(6) Spectrum Files
  |-(1) Minora Feature Detector
  |-(2) Spectrum Selector
   |-(3) Precursor Detector
    |-(21) Sequest HT
     |-(5) Percolator

Processing node 6: Spectrum Files

Input Data:
- File Name(s): H:\ToxinScorpion Said\BOT_G50_digest_nodilution_1µL.raw
----
Processing node 1: Minora Feature Detector
----
1. Storage Settings:
- Feature Traces to Store: All

2. Peak & Feature Detection:
- Min. Trace Length: 5
- S/N Threshold: 1
- Max. ΔRT of Isotope Pattern Multiplets [min]: 0.2

3. Feature to ID Linking:
- PSM Confidence At Least: Medium

Processing node 2: Spectrum Selector
----
1. General Settings:
- Precursor Selection: Use MS1 Precursor
- Use Isotope Pattern in Precursor Reevaluation: True
- Provide Profile Spectra: Automatic
- Spectra to Store: All

2. Spectrum Properties Filter:
- Lower RT Limit: 0
- Upper RT Limit: 0
- First Scan: 0
- Last Scan: 0
- Total Intensity Threshold: 0
- Minimum Peak Count: 1

2.1 Spectrum Properties Filter for DDA Spectra:
- Lowest Charge State: 0

- Highest Charge State: 0
- Min. Precursor Mass: 350 Da
- Max. Precursor Mass: 5000 Da

3. Scan Event Filters:
- MS Order: Is Not MS1
- Min. Collision Energy: 0
- Max. Collision Energy: 1000
- Scan Type: Is Full

4. Peak Filters:
- S/N Threshold (FT-only): 1.5

5. Replacements for Unrecognized Properties:
- Unrecognized Charge Replacements: Automatic
- Unrecognized Mass Analyzer Replacements: ITMS
- Unrecognized MS Order Replacements: MS2
- Unrecognized Activation Type Replacements: CID
- Unrecognized Polarity Replacements: +
- Unrecognized MS Resolution@200 Replacements: 60000
- Unrecognized MSn Resolution@200 Replacements: 30000

6. Precursor Pattern Extraction:
- Precursor Clipping Range Before: 2.5 Da
- Precursor Clipping Range After: 5.5 Da

Processing node 3: Precursor Detector
----
1. General Settings:
- S/N Threshold: 1.5
- S/N Threshold for most abundant peak: 1.5

Processing node 21: Sequest HT
----
1. Input Data:
- Protein Database: ScorpianVariants042326.fasta
- Enzyme Name: Trypsin (Full)
- Max. Missed Cleavage Sites: 3
- Min. Peptide Length: 6
- Max. Peptide Length: 150
- Max. Number of Peptides Reported: 10
- Index Creation Mode: Automatic

2. Tolerances:
- Precursor Mass Tolerance: 10 ppm
- Fragment Mass Tolerance: 0.05 Da
- Use Average Precursor Mass: False
- Use Average Fragment Mass: False

3. Spectrum Matching:
- Use Neutral Loss a Ions: True
- Use Neutral Loss b Ions: True
- Use Neutral Loss y Ions: True
- Use Flanking Ions: True
- Weight of a Ions: 0
- Weight of b Ions: 1
- Weight of c Ions: 0
- Weight of x Ions: 0
- Weight of y Ions: 1
- Weight of z Ions: 0

4. Dynamic Modifications:
- Max. Equal Modifications Per Peptide: 3
- Max. Dynamic Modifications Per Peptide: 4
- 1. Dynamic Modification: Carbamidomethyl / +57.021 Da (C)
- 2. Dynamic Modification: Oxidation / +15.995 Da (C, F, M, S, T, W)
- 3. Dynamic Modification: Phospho / +79.966 Da (S, T, Y)
- 4. Dynamic Modification: Deamidated / +0.984 Da (N, Q, R)

- 5. Dynamic Modification: Pro->Pyrrolidinone / -30.011 Da (P)
- 6. Dynamic Modification: Methyl / +14.016 Da (C, D, E, H, I, K, L, N, Q, R, S, T)

Processing node 5: Percolator
----
1. Target/Decoy Strategy:
- Target/Decoy Selection: Concatenated
- Validation Based on: q-Value

2. Input Data:
- Maximum Delta Cn: 0.05
- Maximum Rank: 0

3. FDR Targets:
- Target FDR (Strict): 0.01
- Target FDR (Relaxed): 0.05

Consensus Step: Workflow
Result name: BOT_G50_digest_nodilution_1µL
Result file: C:\Users\ORBIT ANALYSIS\Master\ProteomeDiscovererResults\ProteomeDiscovererVaraints042426\BOT_G50_digest_nodilution_1µL.pdResult
Description: Result filtered for high confident peptides and high confident master proteins, with enhanced peptide and protein annotations. Add FASTA file with common contaminants to the Protein Marker node. Quan abundances are normalized to the same total peptide amount per channel and scaled, so that the average abundance per protein and peptide is 100.
Workflow based on template: CWF_Comprehensive_Enhanced Annotation_LFQ_and_Precursor_Quan_10ppm
Creation date: 4/23/2026 4:47:39 PM
Created with Discoverer version: 3.2.0.450

The workflow tree:

```
  |-(0) MSF Files
    |-(10) Feature Mapper
      |-(11) Precursor Ions Quantifier
    |-(1) PSM Grouper
      |-(2) Peptide Validator
        |-(3) Peptide and Protein Filter
          |-(4) Protein Marker
          |-(6) Protein Scorer
            |-(7) Protein Grouping
              |-(8) Peptide in Protein Annotation
              |-(9) Protein FDR Validator
```

Post-processing nodes:

```
  |-(12) Result Statistics
  |-(13) Data Distributions
  |-(14) Display Settings
  |-(15) Statistics Insights
```

Processing node 0: MSF Files
----
1. Storage Settings:
- Spectra to Store: All
- Feature Traces to Store: All

2. Merging of Identified Peptide and Proteins:
- Merge Mode: Globally by Search Engine Type

3. FASTA Title Line Display:
- Reported FASTA Title Lines: Best match
- Title Line Rule: standard

4. PSM Filters:
- Maximum Delta Cn: 0.05
- Maximum Rank: 0
- **Maximum Delta Mass: 10 ppm**

Hidden Parameters:
- MSF File(s): H:\ToxinScorpion Said\Variants042326\BOT_G50_digest_nodilution_1µL.msf

----
Processing node 10: Feature Mapper
----
1. Chromatographic Alignment:
- Perform RT Alignment: True
- Parameter Tuning: Coarse
- Maximum RT Shift [min]: 10
- Mass Tolerance: 10 ppm

2. Feature Linking and Mapping:
- RT Tolerance [min]: 0
- Mass Tolerance: 0 ppm
- Min. S/N Threshold: 5
- Map single PSMs: True

Processing node 11: Precursor Ions Quantifier
----
1. General Quantification Settings:
- Peptides to Use: Unique + Razor
- Consider Protein Groups for Peptide Uniqueness: True
- Use Shared Quan Results: False
- Reject Quan Results with Missing Channels: False

2. Precursor Quantification:
- Precursor Abundance Based On: Intensity
- Min. # Replicate Features [%]: 0

3. Normalization and Scaling:
- Normalization Mode: Total Peptide Amount
- Scaling Mode: On All Average

4. Exclude Peptides from Protein Quantification:
- For Normalization: Use All Peptides
- For Protein Roll-Up: Use All Peptides
- For Pairwise Ratios: Exclude Modified

5. Quan Rollup and Hypothesis Testing:
- Protein Abundance Calculation: Summed Abundances
- N for Top N: 3
- Protein Ratio Calculation: Pairwise Ratio Based
- Maximum Allowed Fold Change: 100
- Imputation Mode: None
- Hypothesis Test: t-test (Background Based)
- Calculate Hypothesis Test for Peptides: False

6. Quan Ratio Distributions:
- 1st Fold Change Threshold: 2
- 2nd Fold Change Threshold: 4
- 3rd Fold Change Threshold: 6
- 4th Fold Change Threshold: 8
- 5th Fold Change Threshold: 10

Processing node 1: PSM Grouper
----
1. Peptide Group Modifications:
- Site Probability Threshold: 75

2. Table Creation and Display:
- Automatic Peptide Table Creation: True
- Additional Peptide Entity Creation: None

Processing node 2: Peptide Validator
----
1. General Validation Settings:
- Validation Mode: Automatic (Control peptide level error rate if possible)
- Target FDR (Strict) for PSMs: 0.01
- Target FDR (Relaxed) for PSMs: 0.05
- Target FDR (Strict) for Peptides: 0.01
- Target FDR (Relaxed) for Peptides: 0.05

2. Specific Validation Settings:
- Validation Based on: q-Value
- Target/Decoy Selection for PSM Level FDR Calculation Based on Score: Automatic
- Reset Confidences for Nodes without Decoy Search (Fixed score thresholds): False

----
Processing node 3: Peptide and Protein Filter
----
1. Peptide Filters:
- Peptide Confidence At Least: High
- Keep Lower Confident PSMs: False
- Minimum Peptide Length: 6
- Remove Peptides Without Protein Reference: False

2. Protein Filters:
- Minimum Number of Peptide Sequences: 1
- Count Only Rank 1 Peptides: False
- Count Peptides Only for Top Scored Protein: False

Processing node 4: Protein Marker
----
5. Annotate Species:
- As Species Map: False
- As Species Names: False

6. Mark Additional Entities:
- Annotation Groups: False
- Pathway Groups: False
- Modification Sites: True
- Peptide Isoform Groups: True
- DIA Precursor Groups: True
- DIA Precursors: True
- Peptide Sequence Groups: True

Processing node 6: Protein Scorer

No parameters

Processing node 7: Protein Grouping
----
1. Protein Grouping:
- Apply strict parsimony principle: True

Processing node 8: Peptide in Protein Annotation
----
1. Flanking Residues:
- Annotate Flanking Residues of the Peptide: True
- Number Flanking Residues in Connection Tables: 1

2. Modifications in Peptide:
- Protein Modifications Reported: Only for Master Proteins

3. Modifications in Protein:
- Modification Sites Reported: All And Specific
- Minimum PSM Confidence: High

- Report Only PTMs: True

4. Positions in Protein:
- Protein Positions for Peptides: Only for Master Proteins

Processing node 9: Protein FDR Validator
----
1. Confidence Thresholds:
- Target FDR (Strict): 0.01
- Target FDR (Relaxed): 0.05

Processing node 12: Result Statistics

No parameters

Processing node 13: Data Distributions
----
1. ID Distributions (Bottom-up):
- Peptides to Use: Only unique peptides based on protein groups

----
Processing node 14: Display Settings
----
1. General:
- Filter Set:

```
	###	This file contains the following filters:
	###
	###	Row Filter for Proteins:
	###	-----------------------------------
	###	AND
	###	|
	###	+--Master is equal to Master
	###	|
	###	+--in category
	###	   |
	###	   +--Protein FDR Confidence
	###	   |
	###	   +--High
	###	-----------------------------------
	###
	###
	###	Row Filter for Input Files:
	###	-----------------------------------
	###	FileName does not end with msf
	###	-----------------------------------
	###
```

'magellan filter set' 1 'HighConfidentMasterProteinFilter.filterset' FiltersetProperties 1 'LastFileName' 'Z:\HighConfidentMasterProteinFilter.filterset' Filter 'AminoAcids' FilterProperties 1 'Filter/DisplayPropertyHint' 'Amino Acids' 2 TRUE FilterConditionProperties 1 'PredefinedProperty' 'ExcludedBy' TRUE Filter 'DecoyPeptideGroup' FilterProperties 1 'Filter/DisplayPropertyHint' 'Decoy Peptide Groups' 2 TRUE FilterConditionProperties 1 'PredefinedProperty' 'ExcludedBy' = property 'System.Int32, mscorlib' 'ExcludedBy' constant 'System.Int32, mscorlib' '-1' Filter 'DecoyPeptideSpectrumMatch' FilterProperties 1 'Filter/DisplayPropertyHint' 'Decoy PSMs' 2 TRUE FilterConditionProperties 1 'PredefinedProperty' 'ExcludedBy' = property 'System.Int32, mscorlib' 'ExcludedBy' constant 'System.Int32, mscorlib' '-1' Filter 'DecoyProtein' FilterProperties 1 'Filter/DisplayPropertyHint' 'Decoy Proteins' 2 TRUE FilterConditionProperties 1 'PredefinedProperty' 'ExcludedBy' = property 'System.Int32, mscorlib' 'ExcludedBy' constant 'System.Int32, mscorlib' '-1' Filter 'DecoyProteinGroup' FilterProperties 1 'Filter/DisplayPropertyHint' 'Decoy Protein Groups' 2 TRUE FilterConditionProperties 1 'PredefinedProperty' 'ExcludedBy' = property 'System.Int32, mscorlib' 'ExcludedBy' constant 'System.Int32, mscorlib' '-1' Filter 'DecoyProteoformSpectrumMatch' FilterProperties 1 'Filter/DisplayPropertyHint' 'Decoy PrSMs' 2 TRUE FilterConditionProperties 1 'PredefinedProperty' 'ExcludedBy' = property 'System.Int32, mscorlib' 'ExcludedBy' constant 'System.Int32, mscorlib' '-1' Filter 'TargetProtein' FilterProperties 1 'Filter/DisplayPropertyHint' 'Proteins' 2 NARY_AND 2 = FilterConditionProperties 1 'NamedComparableFilterCondition/DisplayPropertyHint' 'Master' property 'Thermo.PD.EntityDataFramework.MasterProteinAssessment, Thermo.Magellan.EntityDataFramework' 'IsMasterProtein' constant 'Thermo.PD.EntityDataFramework.MasterProteinAssessment, Thermo.Magellan.EntityDataFramework' 'IsMasterProtein' datadistribution_particular FilterConditionProperties 1 'NamedComparableFilterCondition/DisplayPropertyHint' 'Protein FDR Confidence' property

'Thermo.Magellan.EntityDataFramework.DataDistributionValue, Thermo.Magellan.EntityDataFramework' 'ProteinFDRConfidence' constant 'Thermo.Magellan.EntityDataFramework.DataDistributionValue, Thermo.Magellan.EntityDataFramework' 'position_id_1' Filter '7899A5F8-6954-4E95-8C2D-BB0C83221B21' 1 datadistribution_level_equals property 'System.Nullable`1[[System.Int32, mscorlib]], mscorlib' '6AED4774-1ECD-4202-B746-B79D936A3D77' constant 'System.Nullable`1[[System.Int32, mscorlib]], mscorlib' '4' FilterConditionProperties 1 'PredefinedProperty' 'ExcludedBy' = property 'System.Int32, mscorlib' 'ExcludedBy' constant 'System.Int32, mscorlib' '-1' Filter 'TargetProteoformSpectrumMatch' FilterProperties 1 'Filter/DisplayPropertyHint' 'PrSMs' 2 TRUE FilterConditionProperties 1 'PredefinedProperty' 'ExcludedBy' = property 'System.Int32, mscorlib' 'ExcludedBy' constant 'System.Int32, mscorlib' '-1' Filter 'UnknownModification' FilterProperties 1 'Filter/DisplayPropertyHint' 'Unknown Modifications' 2 TRUE FilterConditionProperties 1 'PredefinedProperty' 'ExcludedBy' TRUE Filter 'WorkflowInputFile' FilterProperties 1 'Filter/DisplayPropertyHint' 'Input Files' 2 NARY_AND 1 endsnotwith property 'System.String, mscorlib' 'FileName' constant 'System.String, mscorlib' 'msf' FilterConditionProperties 1 'PredefinedProperty' 'ExcludedBy' TRUE

Processing node 15: Statistics Insights
----
1. User Statistics:
- Stats Configuration: noDIA_stats

User Defined Filters:
- User Filter Set 1: HighConfident_ProteinGroups
- User Filter Set 2: HighConfident_Proteins
- User Filter Set 3: HighConfident_PeptideGroups
- User Filter Set 4: HighConfident_PSMs

Supplementary Data S2 casanovo parameter:

MTD	mzTab-version	1.0.0
MTD	mzTab-mode	Summary
MTD	mzTab-type	Identification
MTD	description	Casanovo identification file predictions
MTD	software[1]	[MS, MS:1003281, Casanovo, 5.1.2]
MTD	psm_search_engine_score[1]	[MS, MS:1001143, search engine specific score for PSMs,]
MTD	fixed_mod[1]	[MS, MS:1002453, No fixed modifications searched,]
MTD	variable_mod[1]	[MS, MS:1002454, No variable modifications searched,]
MTD	software[1]-setting[1]	model = C:\Users\ AppData\Local\casanovo\casanovo_v5_0_0_v5_0_0.ckpt
MTD	software[1]-setting[2]	config_filename = default
MTD	software[1]-setting[3]	precursor_mass_tol = 50.0
MTD	software[1]-setting[4]	isotope_error_range = (0, 1)
MTD	software[1]-setting[5]	min_peptide_len = 6
MTD	software[1]-setting[6]	max_peptide_len = 100
MTD	software[1]-setting[7]	predict_batch_size = 1024
MTD	software[1]-setting[8]	top_match = 1
MTD	software[1]-setting[9]	accelerator = auto
MTD	software[1]-setting[10]	devices = None
MTD	software[1]-setting[11]	n_beams = 1
MTD	software[1]-setting[12]	enzyme = trypsin
MTD	software[1]-setting[13]	digestion = full
MTD	software[1]-setting[14]	missed_cleavages = 0
MTD	software[1]-setting[15]	max_mods = 1
MTD	software[1]-setting[16]	allowed_fixed_mods = C:C[Carbamidomethyl]
MTD	software[1]-setting[17]	allowed_var_mods = M:M[Oxidation],N:N[Deamidated],Q:Q[Deamidated],nterm:[Acetyl]-,nterm:[Carbamyl]-,nterm:[Ammonia-loss]-,nterm:[+25.980265]-
MTD	software[1]-setting[18]	random_seed = 454
MTD	software[1]-setting[19]	n_log = 1
MTD	software[1]-setting[20]	tb_summarywriter = False
MTD	software[1]-setting[21]	log_metrics = False

MTD	software[1]-setting[22]	log_every_n_steps = 50
MTD	software[1]-setting[23]	lance_dir = None
MTD	software[1]-setting[24]	val_check_interval = 50000
MTD	software[1]-setting[25]	min_peaks = 20
MTD	software[1]-setting[26]	max_peaks = 150
MTD	software[1]-setting[27]	min_mz = 50.0
MTD	software[1]-setting[28]	max_mz = 2500.0
MTD	software[1]-setting[29]	min_intensity = 0.01
MTD	software[1]-setting[30]	remove_precursor_tol = 2.0
MTD	software[1]-setting[31]	max_charge = 4
MTD	software[1]-setting[32]	dim_model = 512
MTD	software[1]-setting[33]	n_head = 8
MTD	software[1]-setting[34]	dim_feedforward = 1024
MTD	software[1]-setting[35]	n_layers = 9
MTD	software[1]-setting[36]	dropout = 0.0
MTD	software[1]-setting[37]	dim_intensity = None
MTD	software[1]-setting[38]	warmup_iters = 100000
MTD	software[1]-setting[39]	cosine_schedule_period_iters = 600000
MTD	software[1]-setting[40]	learning_rate = 0.0005
MTD	software[1]-setting[41]	weight_decay = 1e-05
MTD	software[1]-setting[42]	train_label_smoothing = 0.01
MTD	software[1]-setting[43]	train_batch_size = 32
MTD	software[1]-setting[44]	max_epochs = 30
MTD	software[1]-setting[45]	shuffle = True
MTD	software[1]-setting[46]	shuffle_buffer_size = 10000
MTD	software[1]-setting[47]	num_sanity_val_steps = 0
MTD	software[1]-setting[48]	calculate_precision = False
MTD	software[1]-setting[49]	accumulate_grad_batches = 1
MTD	software[1]-setting[50]	gradient_clip_val = None
MTD	software[1]-setting[51]	gradient_clip_algorithm = None
MTD	software[1]-setting[52]	precision = 32-true
MTD	software[1]-setting[53]	replace_isoleucine_with_leucine = True
MTD	software[1]-setting[54]	massivekb_tokenizer = False
MTD	software[1]-setting[56]	n_workers = 0

Supplementary Figure S1

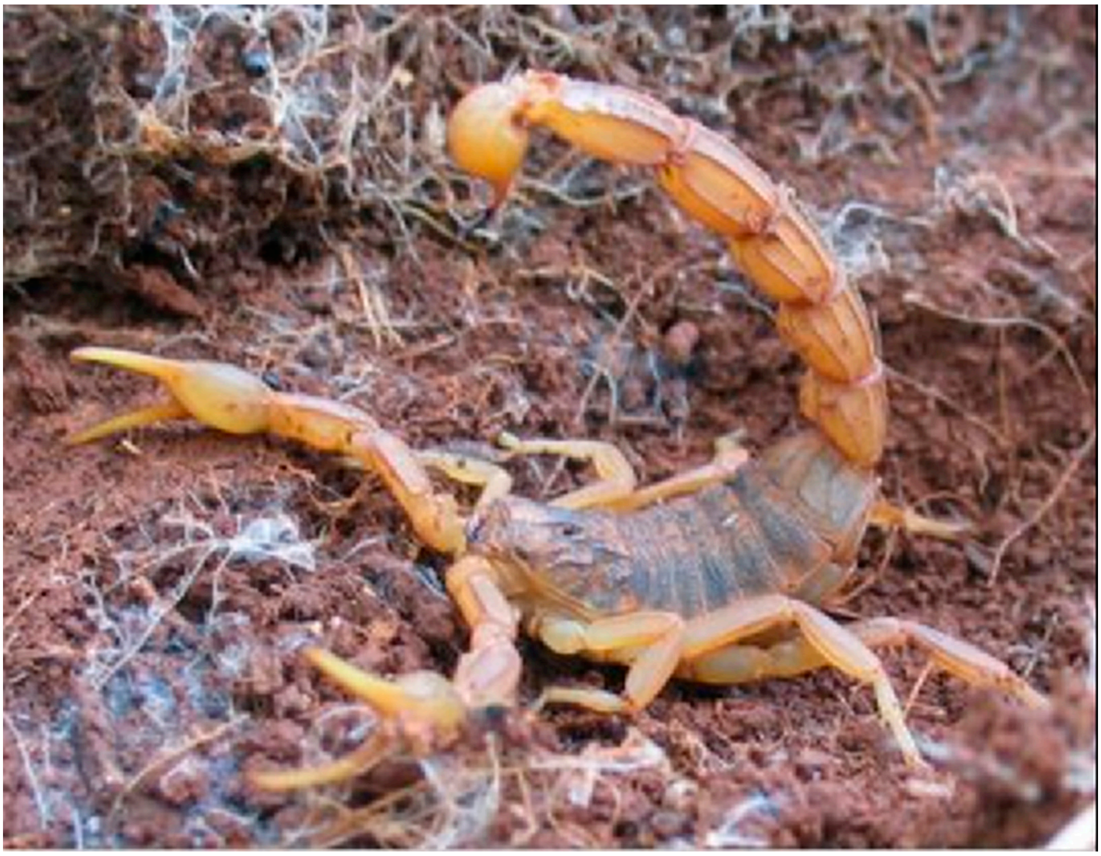

Figure S1. Representative habitus of a *Buthus* specimen attributed to *Buthus tunetanus*. The photograph illustrates the general colour pattern, pedipalps, metasoma, telson and aculeus. It is provided for contextual taxonomic documentation and should not be interpreted as a formal morphological redetermination of the exact individual specimens that produced the archived pooled venom. The absence of standardized diagnostic views, complete morphometric examination and voucher specimens is explicitly acknowledged.
